# From Marine Metabolites to the Drugs of the Future: Squalamine, Trodusquemine, Their Steroid and Triterpene Analogues †

**DOI:** 10.3390/ijms23031075

**Published:** 2022-01-19

**Authors:** Oxana Kazakova, Gulnara Giniyatullina, Denis Babkov, Zdenek Wimmer

**Affiliations:** 1 Ufa Institute of Chemistry, UFA Federal Research Centre of the Russian Academy of Sciences, Pr. Oktyabrya, 450054 Ufa, Russia; gulnaravlg@gmail.com; 2 Laboratory of Metabotropic Drugs, Scientific Center for Innovative Drugs, Volgograd State Medical University, Novorossiyskaya St. 39, 400087 Volgograd, Russia; denis.a.babkov@gmail.com; 3 Department of Chemistry of Natural Compounds, University of Chemistry and Technology in Prague, Technicka’ 5, Prague 6, 16628 Prague, Czech Republic; Zdenek.Wimmer@vscht.cz

**Keywords:** squalamine, trodusquemine, ceragenine, claramine, triterpenoids, antibiotic, angiogenesis, obesity, diabetes

## Abstract

This review comprehensively describes the recent advances in the synthesis and pharmacological evaluation of steroid polyamines squalamine, trodusquemine, ceragenins, claramine, and their diverse analogs and derivatives, with a special focus on their complete synthesis from cholic acids, as well as an antibacterial and antiviral, neuroprotective, antiangiogenic, antitumor, antiobesity and weight-loss activity, antiatherogenic, regenerative, and anxiolytic properties. Trodusquemine is the most-studied small-molecule allosteric PTP1B inhibitor. The discovery of squalamine as the first representative of a previously unknown class of natural antibiotics of animal origin stimulated extensive research of terpenoids (especially triterpenoids) comprising polyamine fragments. During the last decade, this new class of biologically active semisynthetic natural product derivatives demonstrated the possibility to form supramolecular networks, which opens up many possibilities for the use of such structures for drug delivery systems in serum or other body fluids.

## 1. Introduction

Biogenic polymethylene polyamines are found in all living cells in significant quantities and are involved in many important biological processes [1,2]. The biosynthetic pathways to these polyamines in animals, plants, and microorganisms are well known and originate from amino acids. In addition to the simplest form as free aliphatic bases, they are often found as structural units of numerous alkaloids of plant and animal origin, which are usually referred to as secondary metabolites [3,4].

The study of metabolites of marine organisms in the second half of the last century became a separate major area of bioorganic and medicinal chemistry, influencing the development of synthetic organic chemistry. In a long line of marine metabolites, striking in the diversity and complexity of their structures, polyamine compounds occupy the main place. In contrast to the plant metabolites, which are mainly derived from putrescine, spermidine, and spermine, the metabolites of marine organisms are much more diverse structurally [5].

**Figure 1:**
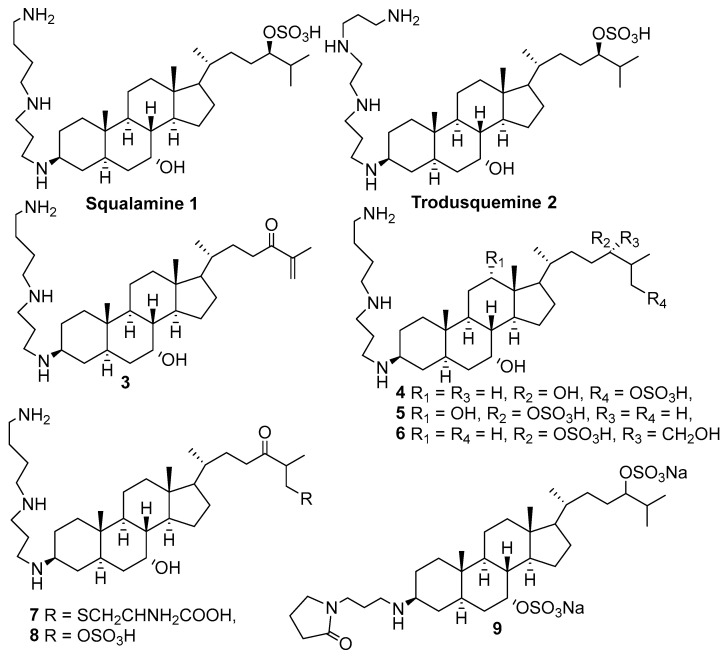
The structures of squalamine 1, trodusquemine 2 and steroid polyamines 3–9.

The history of steroid polyamines began in the early 1990s with the isolation by Moore et al. of the first representative compound squalamine 1 from the stomach of the shark *S. acanthias* (Figure 1) [6]. The name “squalamine” originated from the Latin “Squalus”, the genus name for the shark. The team leader, Prof. Zasloff, has long been intrigued in shark endurance and immunity to infections: when a shark is operated on, you do not have to worry about aseptic conditions, despite the fact that the immune system of sharks, especially a sea dog, is poorly developed. Squalamine was also isolated from other organs of the shark (spleen, intestines, ovaries) [7,8,9], its maximum content was noted in the liver and gallbladder. In addition, it was identified in the blood cells of the sea lamprey *P. marinus* [10]. Squalamine was later found to directly protect sharks from infections via broad-spectrum antibiotic activity [4,5,6,7,11,12,13]. In 1998, Sills et al. showed that squalamine effectively inhibits angiogenesis and tumor growth in several animal models [14]. Recently, squalamine proved effective as neuroprotective agent in animal model of Parkinson’s disease [15].

The chemical structure of squalamine 1 as 3β-*N*-1-(*N*-[3-(4-aminobutyl)]- 1,3-diaminopropane)-7α,24ζ-dihydroxy-5α-cholestan-24-sulfate was determined by methods of mass and NMR spectroscopy [6].

Later, seven other amino sterols 2–8 (Figure 1) with antibacterial activity, structurally similar to squalamine, were isolated from the liver of the shark *S. acanthias* [3]. They contain a cholestan skeleton conjugated to spermidine or spermine at the C3 position, while the side chain can be sulfated. One of them, trodusquemine (MSI-1436) 2, also has a broad spectrum of antimicrobial activity, even slightly surpassing squalamine [6,7]. Petromysonamine disulfate 9 was isolated from sea lamprey pheromone *P. marines* [16,17]. The authors of [16] suggest that squalamine 1 can be a biosynthetic precursor of PADS [18]. Subsequently, the 24*S*-epimer was synthesized from aldehyde-derived 24-methanal-7α-(methoxymethyl)cholestan-3-one [16].

As a result of these studies, squalamine became the first representative of a previously unknown class of natural antibiotics of animal origin, and its discovery stimulated extensive research on the complete synthesis, creation of various libraries of steroid polyamines, and the study of their properties. The interest shown in the chemistry and pharmacology of squalamine, trodusquemine, and their analogs is supported by thousands of patent applications that have been filed to date. Aminosterols have proven to be promising chemotherapeutic agents for the treatment of infectious and neoplastic diseases. Subsequently, the known biological activity of this class of compounds was significantly expanded and continues to be supplemented annually. Besides a promising antimicrobial activity [4] squalamine 1, trodusquemine 2, and their analogues 3–9 have been proposed for ameliorating blood pressure [19], treatment of Alzheimer’s disease [20], Parkinson’s disease [21], constipation [22], erectile dysfunction [23], cardiac conduction defects [24], cognitive impairment [25], autism spectrum disorder [26], multiple system atrophy [27], depression [28], schizophrenia [29] and viral infections [30].

Despite the fact that many works have been devoted to the chemistry and pharmacology of steroid polyamines, including reviews [11,12,31,32,33,34,35,36,37,38,39,40,41,42], until now the scientific literature has not summarized information on complete syntheses, various modifications and biological activity of steroid polyamines. The goal of this review is to systematize the information available in the literature (up to 2021 with impact up to the most recent years) on the chemistry and biological activity of steroid polyamines, including their influence on the synthesis, properties, and perspectives of terpenoids with polyamine fragments.

## 2. Syntheses of Squalamine from Cholic Acids

Due to the need for significant amounts of squalamine for biological tests, schemes for its synthesis from available steroids (3β-acetoxy-5-cholic acid, 3-keto-23,24-bisnorchol-4-en-22-ol, methyl-3-keto-5α-chenodeoxycholonate) were implemented [8,9,43]. The maximum overall yield (69%) was achieved in a 7-step synthesis by the reaction of reductive amination of 24*R*-sulfate of 3-ketocholesterol with azide and further catalytic hydrogenolysis [44]. The syntheses of squalamine are discussed below.

### 2.1. Synthesis Based on 3β-Acetoxy-5-Cholic Acid

**Scheme 1:**
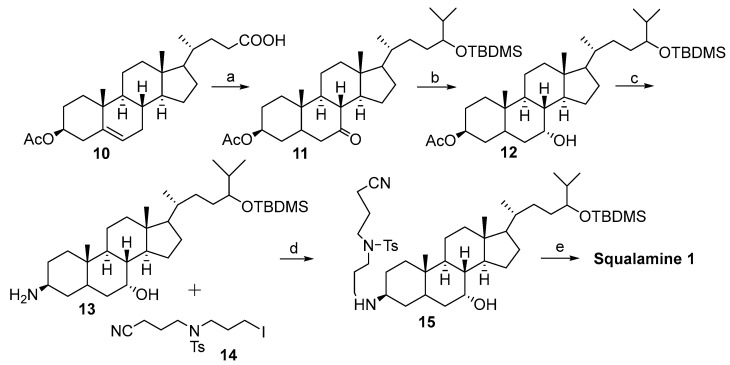
Reagents and conditions: (**a**) 1. (COCl)_2_, CH_2_Cl_2_, 40 °C, 2 h; 2. (CH_3_)_2_CHCdBr, benzene, 25 °C, 1 h; 3. Ca(BH_4_)_2_, THF, 25 °C, 5 h; 4. TBDMsCl, imidazol, CH_2_Cl_2_, 16 °C, 16 h; 5. Cr(CO)_6_, *t*-BuOOH, CH_3_CN, 50 °C, 12 h; 6. Li, NH_3_, Et_2_O, −78 °C, 10 min; (**b**) 1. K-selectride, THF, −50 °C, 5 h; 2. NaCN, MeOH, 60 °C, 8 h; 3. (*t-*BuO)_3_Al, hexane, toluene, 110 °C, 20 h; (**c**) 1. C_6_H_5_CH_2_ONH_2_ HCl, Py, 115 °C, 16 h; 2. LiAlH_4_, Et_2_O, 35 °C, 16 h; (**d**) K_2_CO_3_, CH_3_CN, 50 °C, 20 h; (**e**) 1. C_6_H_5_CH_2_OCOCl, NaOH, THF, 0–25 °C, 4 h; 2. Na, NH_3_, THF, −78 °C, 18 h; 3. LiAlH_4_, Et_2_O, 35 °C, 6 h; 3. HCl, EtOH, 25 °C, 3 h; 4. SO_3_-Py, Py, 75 °C, 2 h.

The first synthesis of squalamine 1 was carried out by Moriarty et al. in 1994 based on 3β-acetoxy-5-cholic acid 10 (Scheme 1) [8,45]. Starting with the protection of the carboxyl group (compound **11**), and then as a result of successive reactions (reduction, deprotection, and oxidation), the keto derivative 12 was obtained, which was converted to 3β-amino-7α-hydroxy-cholestan 13 [46,47,48]. The reaction of compound **13** with tosyl-*N*-(3-cyanopropyl)-*N*-propyl iodide 14 led to 3β-*N*-diaminopropyl-butyronitrile-7α-hydroxy-cholestan 15. After reduction of the cyano group, deprotection, and sulfation of the 24β-hydroxyl group, squalamine 1 was obtained [49,50]. The synthesis included 17 steps with a total yield of 0.3%.

### 2.2. Synthesis of 24R- and 24S-Squalamine’s from Stigmasterol

**Scheme 2:**
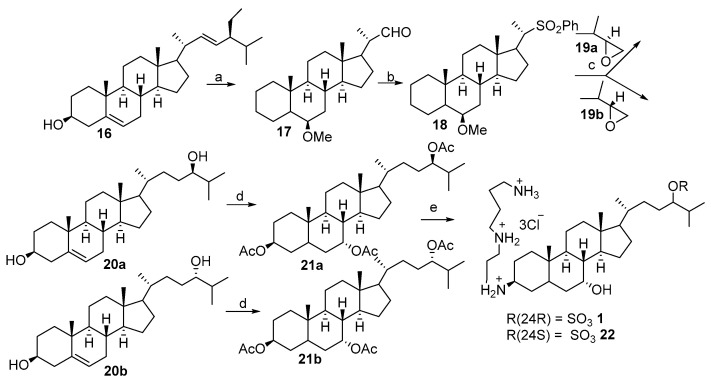
Reagents and conditions: (**a**) 1. TsCl, Py, 14 h, 25 °C; 2. AcOK, MeOH, 4 h, 25 °C; 3. O_3_, MeOH, −78 °C; (**b**) 1. NaBH_4_, MeOH, 0–25 °C; 2. CH_3_SO_2_Cl, Et_3_N, CH_2_Cl_2_, 2 h, 0 °C; 3. NaI, acetone, 17 h, 25 °C; 4. PhSO_2_Na, DMF, 32 h, 25 °C; (**c**) 1. *n*-BuLi, 2 h, −78 °C; 2. Li, NH_3_, 30 min, −78 °C; 3. TsOH, dioxane/H_2_O, (7:3), 1 h, 80 °C; (**d**) 1. Ac_2_O, Py, 14 h, 25 °C; 2. CrO_3_, DMAP, CH_2_Cl_2_, 24 h, −20 °C; 3. Li, NH_3_, 10 min, −78 °C; 4. KB[CH(CH_3_)C_2_H_5_]_3_H, THF, 6 h, −50 °C; 5. Ac_2_O, DMAP, CH_2_Cl_2_, 14 h, 25 °C; (**e**) 1. CrO_3_, H_2_SO_4_, H_2_O, 7 h, 25 °C; 2. BocNH(CH_2_)_4_N(Boc)(CH_2_)_3_NH_2_, NaBH_3_CN, MeOH, 14 h, 25 °C; 3. HCl, MeOH, 11 h, 25 °C; 4. SO_3_-Py, Py, 6 h, 25 °C.

A year later, the same group presented a 19-step synthesis of 24*R*- and 24*S*-squalamine’s from stigmasterol 16 with a total yield of 19% (Scheme 2) [37]. Stigmasterol 16 in several stages (selective Boc-protection, ozonolysis) was converted into aldehyde 17, while its reduction, chlorination of the hydroxy derivative, treatment with sodium phenylsulfone led to phenylsulfone 18. Its interactions with enantiomeric epoxides 19a and 19b afforded 24*R*- and 24*S*-hydroxycholesterols 20a and 20b in quantitative yields. Further acetylation, oxidation of the corresponding diacetates, reduction of the obtained enones with Li/NH_3_, potassium tri-*tert*-butylborohydride and repeated acylation produced *tris*-acetates 21a and 21b, while their selective deacylation at position C3 and oxidation with Jones’s reagent led to 3-ketocholonates. At the last step, reductive amination with Boc-spermidine/NaBH_3_CN and sulfation resulted in 24*R*-1 and 24*S*-22 squalamines.

### 2.3. Synthesis from 7α-(Benzyloxy)-3-Dioxolan-Cholestan-24R-Ol

**Scheme 3:**
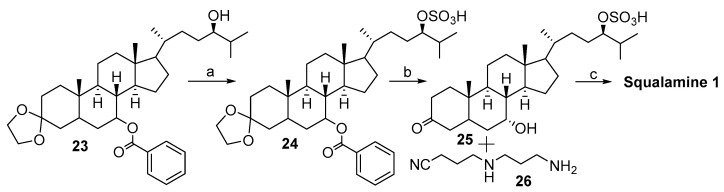
Reagents and conditions: (**a**) 1. Amberlist 15, acetone; 2. SO_3_-Py, Py, 6 h, 80 °C; (**b**) KOH, MeOH, 60 °C; (**c**) 1. NaBH_4_, CH(OCH_3_)_3_, MeOH, −78 °C; 2. Pt_2_O, CF_3_COOH, EtOH.

Zhang et al. in 1998 has carried out a five-step synthesis of squalamine 1 with a total yield of 60% from the readily obtained 7α-(benzyloxy)-3-dioxolan-cholestan-24*R*-ol 23. 24*R*-Sulfate 24 was synthesized, then the protective groups were removed, as a result of reductive amination of ketone 25 with diaminopropylbutyronitrile 26 in the presence of NaBH_4_/CH(OCH_3_)_3_, followed by catalytic hydrogenolysis, squalamine 1 was synthesized (Scheme 3) [51].

### 2.4. Synthesis from Cholic Acid Sulfate

**Scheme 4:**
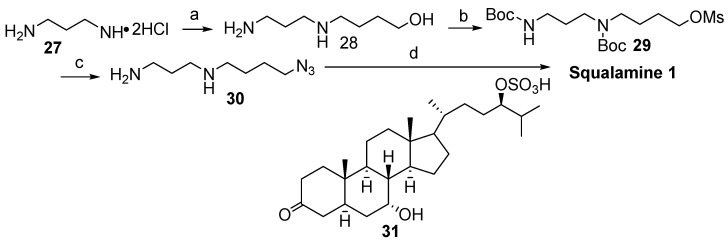
Reagents and conditions: (**a**) 4-chlorobutanol, H_2_O, 140–150 °C; (**b**) 1. Boc_2_O, EtOH, 48 h; 2. Et_3_N, MsCl, CH_2_Cl_2_, 16 h; (**c**) 1. NaN_3_, DMF, 48 h; 2. HCl, dioxane, 16 h; (**d**) 1. 31, NaOMe, MeOH, 24 h, −78 °C, NaBH_4_; 2. H_2_, Ni-Raney.

A year later, Weis et al. put attention to the preparation of the spermidine fragment. The product of the alkylation of 1,3-diaminopropane 27 with chlorobutanol through the protection of amino- to 28 and alcohol groups to 29 was converted into azide 30, which, as a result of the reductive amination of 24*R*-sulfate of 3-ketocholesterol 31 and catalytic hydrogenolysis, was converted into squalamine 1 with a yield of 69% (Scheme 4) [44].

### 2.5. Synthesis from 3-Keto-23,24-Bisnorchol-4-En-22-Ol

**Scheme 5:**
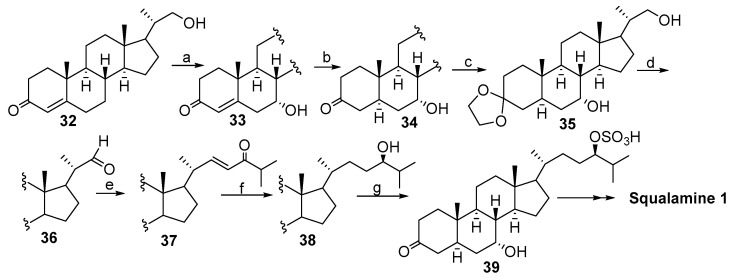
Reagents and conditions: (**a**) *D. gossipina*; (**b**) Li, NH_3_, THF; (**c**) TMSCl, OH(CH_2_)_2_OH; (**d**) NaOCl, TEMPO, NaBr, CH_2_Cl_2_; (**e**) P(O)CH_2_C(O)CH(CH_3_)_2_, Et_2_O, *t*-BuONa, THF; (**f**) 1. (*R*)-MeCBS, BH_3_ THF, toluene; 2. Et_3_N, toluene; 3. H_2_, Pt/C; (**g**) 1. *p*-TsOH, H_2_O, acetone; 2. SO_3_-Py, Py.

In 2000, the same group published a 10-step synthesis of squalamine from 3-keto-23,24-bisnorchol-4-en-22-ol 32 with a total yield of 9% and a purity of 91% (Scheme 5) [9]. Biotransformation of compound **32** in the presence of bacteria *D. gossipina* formed 7α-hydroxy derivative 33. The following steps of preparation of the steroid skeleton included the reduction of the C5(C6) double bond (compound **34**), protection of the C3 ketone (compound **35**), oxidation to aldehyde 36, alkylation of C22 aldehyde with the Wadsworth–Emmons reagent to derivative 37, followed by oxidation with a mixture (*R*)-methyl ester of oxoazaborolidene (MeCBS) with a borane-tetrahydrofuran complex and reduction of 24-ketone to the hydroxy derivative 38. The ethylene ketal protection was removed by the action of *p*-toluenesulfonic acid, and the intermediate 39 was obtained by sulfation of the 24β-hydroxyl group with complex SO_3_-Py in dry pyridine. Reductive amination of 24*R*-sulfate 39 with spermidine and reduction of the resulting Schiff base with sodium cyanoborohydride led to the target squalamine 1 [52].

### 2.6. Synthesis from Methyl 3-Keto-5α-Chenodeoxycholonate

**Scheme 6:**
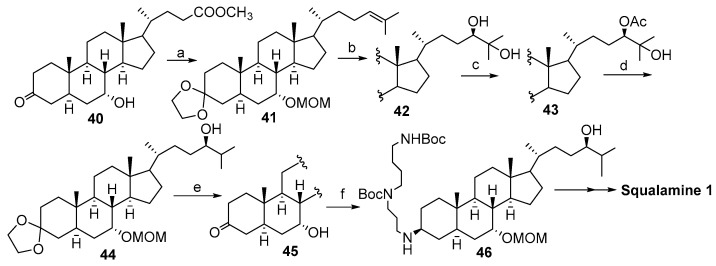
Reagents and conditions: (**a**) 1. CH_3_OCH_2_OCH_3_, P_2_O_5_, CHCl_3_, 25 °C; 2. OH(CH_2_)_2_OH, PTSA, benzene, 70 °C; 3. LiAlH_4_, THF, 25 °C; 4. (COCl)_2_, DMSO, Et_3_N, CH_2_Cl_2_, −78 °C, BuLi, Ph_3_PCH^+^(CH_3_)_2_I^−^, THF; (**b**) (DHQD)_2_PHAL, K_2_OsO_2_(OH)_4_, K_3_Fe(CN)_6_, K_2_CO_3_, CH_3_SO_2_NH_2_, *t*-BuOH/*t*-BuOMe/H_2_O (2.5:3:2.5); (**c**) Ac_2_O, Py; (**d**) 1. CH_3_SO_2_Cl, DMAP, Et_3_N, CH_2_Cl_2_, 0–20 °C; 2. Pd/C, EtOAc, DMF, KOH, MeOH, 95 °C; (**e**) PPTS, *t*-BuOH; (**f**) BocNH(CH_2_)_4_NBoc(CH_2_)_3_NH_2_, NaBH_3_CN, 25 °C.

In 2001 Zhou et al. described the selective synthesis of squalamine 1 from methyl 3-keto-7*α*-chenodeoxycholonate 40 in 11 steps (Scheme 6) [53,54,55]. As in the cases described above, the key steps were aimed at preparing the steroid backbone. The stepwise protection of the 3-keto and 7α-hydroxy groups, chain lengthening of the aldehyde at position C23 by the Wittig reaction led to the desmosterol derivative 41. Its hydroxylation to compound **42**, then dehydration of 24β-acetate 43 and catalytic hydrogenolysis of the isopropenyl group to form compound **44** with following removal of protective groups made 3-keto-24*R*-hydroxy derivative 45, which was introduced into a reductive amination reaction with protected spermidine in the presence of sodium borohydride to give mixtures of 3α- (10%) and 3β-anomers (66%) of squalamine, separated by flash chromatography [50,54,56,57,58,59,60]. The squalamine yield was 19% after removal of the di-*tert*-butyl protecting group in the compound **46** [61].

### 2.7. Synthesis from Desmosterol

**Scheme 7:**
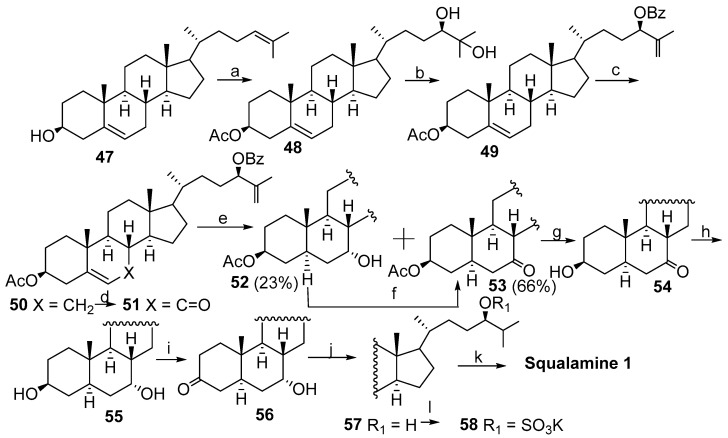
Reagents and conditions: (**a**) 1. Ac_2_O, Py, 110 °C, 4 h; 2. AD-mix-β, *t*-BuOH/H_2_O; 3. BzCl, Py, 0 °C, 24 h; (**b**) POCl_3_, Py, 25 °C, 12 h; (**c**) H_2_, 10% Pd-C, EtOAc, 25 °C, 4 h; (**d**) *N*-hydroxyphthalamide, EtOAc-acetone, C_7_H_5_O_2_, 25 °C, 4 h; (**e**) H_2_, PtO_2_, EtOAc, 25 °C, 3 h; (**f**) PCC, CH_2_Cl_2_, 25 °C, 12 h; (**g**) K_2_CO_3_, MeOH-CHCl_3_, 25 °C, 12 h; (**h**) K[CH(CH_3_)CH_2_CH_3_]_3_BH, THF, H_2_O_2_, −20 °C, 3 h; (**i**) Ag_2_CO_3_-ceolite, toluene, 25 °C, 8 h; (**j**) KOH, HO(CH_2_)_2_OH, 120 °C, 3 h; (**k**) SO_3_-Py, Py, 80 °C, 4 h; (**l**) NaOMe, H_2_N(CH_2_)_3_NH(CH_2_)_4_NH_2_ 2HCl, NaBH_3_CN, H_2_, PtO_2_, 25 °C, 18 h.

In 2003, Okumura et al. synthesized squalamine 1 from desmosterol (Scheme 7) [62]. Pure desmosterol 47 is not a sufficiently available starting compound due to its high cost and low synthesis yields [59]. However, it was found that about 10–25% of desmosterol is contained in the cholesterol precipitate, which is isolated from lanolin alcohol obtained by saponification of animal fat. As in the case of compound **42** (Scheme 6), regioselective protection of diol 48 was carried out in the side chain, its dehydration to compound **49**, and catalytic hydrogenolysis to 24*R*-benzoate 50. Ketone 51 obtained by allyl oxidation was hydrogenated over Adams’ catalyst to give a mixture of 7β-hydroxy and 7-oxo derivatives 52 and 53 [51,63,64]. Stereoselective reduction of ketone 54 with potassium tri-*tert*-butylborohydride led to the dihydroxy derivative 55, its regioselective oxidation with Ag_2_CO_3_ on zeolite was converted into the 3-keto derivative 56 with subsequent replacement of the 24*R*-benzoyl group by the sulfate group through the formation of 24-hydroxy derivative 57. The potassium salt of 3-oxo-7α-hydroxy-24*R*-sulfate-cholanic acid 58 was subjected to reductive amination with spermidine in the presence of NaBH_3_CN to form squalamine 1 in a total yield of 7.4% [62].

### 2.8. Synthesis from Methylhyodeoxycholonate

**Scheme 8:**
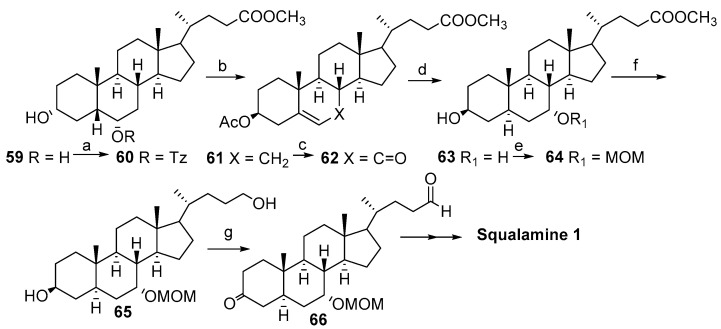
Reagents and conditions: (**a**) TsCl, Py; (**b**) 1. KOAc, H_2_O, DMF; 2. Ac_2_O-Py; (**c**) PDC, TBHP, benzene; (**d**) 1. Pd/C, H_2_; 2. *L*-selectride, THF; (**e**) MOMCl, *i*-Pr_2_NEt, CH_2_Cl_2_; (**f**) LiAlH_4_, THF; (**g**) CrO_3_, Py, CH_2_Cl_2_.

In 2006, Shen et al. proposed a selective 15-step synthesis of squalamine 1 based on methylhyodeoxycholonate 59 with a total yield of 5.6% (Scheme 8) [65]. The main stages in the preparation of the steroid skeleton included protection of the hydroxy group in the C6 position with tosyl chloride to compound **60**, selective hydrolysis, acylation to acetate 61, and oxidation to ketone 62. Subsequent hydrogenation and reduction led to methyl 3β-acetoxy-5α*H*-7α-chenodeoxycholonate 63, which was converted to 24-aldehyde 66 through the steps of protecting compound **64** and extending the C24 side chain to form dihydroxy derivative 66. Further reductive amination with spermidine in the presence of sodium cyanoborohydride, sulfation, and deprotection led to squalamine 1.

Thus, the presented syntheses of squalamine included mainly the preparation the steroid backbone by the modifying of cholic acid scaffolds, obtaining a spermidine fragment, unblocking various protective groups, and sulfating the C24 position. In the following sections, we will consider the syntheses of squalamine analogs.

## 3. Syntheses of Squalamine Analogs

### 3.1. Synthesis of 3α-Episqualamine

**Scheme 9:**
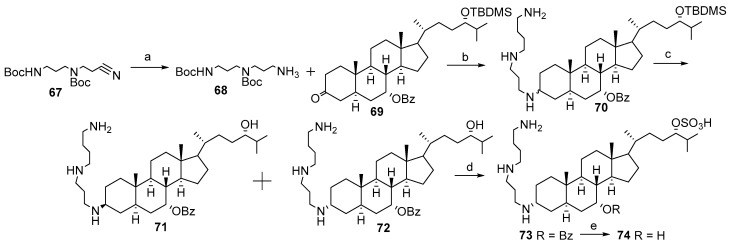
Reagents and conditions: (**a**) LiAlH_4_, Et_2_O, 3Å, 12 h, 0–25 °C; (**b**) NaBH_3_CN, MeOH, 24 h, 25 °C; (**c**) CF_3_COOH, CHCl_3_, 25 °C; (**d**) SO_3_-Py, Py, 114 °C; (**e**) KOH, MeOH, 7 h, 60 °C.

The 3-episqualamine 74, being an 3α-analog of squalamine, was not found in nature, but was synthesized by the reductive amination reaction of Boc-protected spermidine 68, which was obtained from nitrile 67 and 7α-benzyloxy-24ζ-*t*-butyldimethylsilyloxycholestan-3-one 69 (Scheme 9) [66]. As a result of the reaction, a mixture of isomers 70 was obtained; after removal of the protective group a mixture of 3α- 71 and 3β- 72 isomers in equal proportions was formed and separated chromatographically. Sulfation to compound **73**, and subsequent deprotection resulted in 3α-episqualamine 74 in 67% overall yield.

### 3.2. Synthesis of Squalamine Analogs from Cholic Acids

**Scheme 10:**
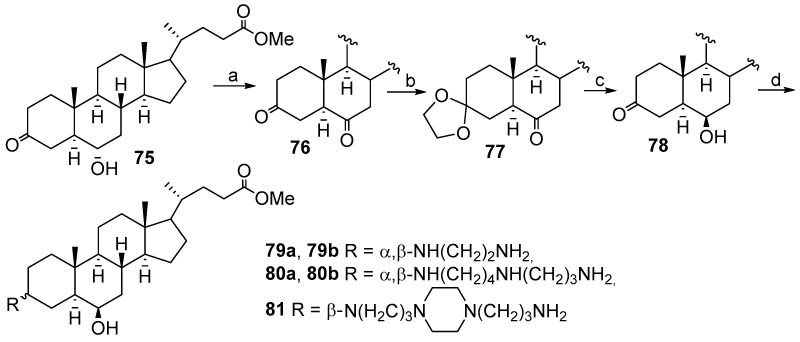
Reagents and conditions: (**a**) PCC, CH_2_Cl_2_; (**b**) HO(CH_2_)_2_OH, TsOH, benzene; (**c**) 1. NaBH_4_, MeOH; 2. HCl, acetone; (**d**) 1. NaBH_3_CN, NH_2_(CH_2_)_2_NH_2_, NH_2_(CH_2_)_4_NH(CH_2_)_3_NH_2_, *N*,*N*-NH_2_(CH_2_)_3_NC_2_H_4_N(CH_2_)_3_NH_2_, THF, MeOH; 2. NaOH, THF.

Methyl 3α,6α-dihydroxycholate 75 was prepared in several steps (oxidation to 3,6-diketone 76, selective dioxolane protection of the C3 position of compound **77**, reduction of the C6 ketone, and deprotection to methyl 3-oxo-6β-hydroxy-5α-cholan-24-oat 78) [67]. Reductive amination of ketone 78 with ethylenediamine or spermidine made it possible to obtain conjugates 79a, 80a and 79b, 80b in 78–82% yields (Scheme 10). In the case of reductive amination with *N*,*N*′-dipropylaminopiperazine, a single 3β-isomer 81 was formed [66].

**Scheme 11:**
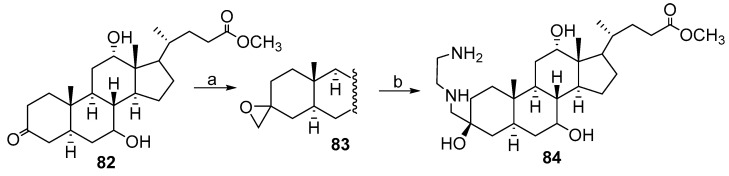
Reagents and conditions: (**a**) trimethylsulfoxonium iodide, NaH, DMSO–THF, 2 h; (**b**) 1. NH_2_(CH_2_)_2_NHBoc, MeOH, 64 °C, 2 h; 2. 50% CF_3_COOH/CH_2_Cl_2_; 3. 50% DIPEA/CH_2_Cl_2_.

The reaction of methyl 3-oxo-cholate 82 with sulfur ylide (trimethylsulfoxonium iodide/NaH) led to 3β-oxirane 83, the nucleophilic opening of which with *N*-(Boc)-1,2-diaminoethane followed by a deprotection led to the compound **84** (Scheme 11) [68].

### 3.3. Synthesis of Steroid Methylenepolyamines from Cholic, Deoxycholic, Chenodeoxycholic, Ursodeoxycholic and Lithocholic Acids

**Scheme 12:**
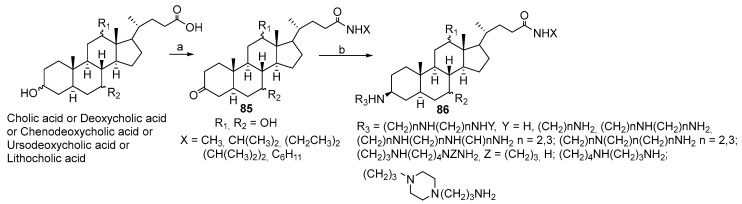
Reagents and conditions: (**a**) 1. HOBT/DCC/BOP/ methyl chloroformate, CHC1_2_/THF/dioxane, −20 °C to 20 °C; 2. aluminum tri-*tert*-butoxide/aluminum triisopropoxide/Ag_2_CO_3_, benzene, toluene, cyclohexane, trifluorotoluene; (**b**) 1. RNH_2_, Ti(O*i*Pr)_4_, MeOH, 20 °C, 12 h; 2. NaBH_4_, MeOH, −78 °C, 2 h.

On the basis of cholic, deoxycholic, lithocholic, chenodeoxycholic, and ursodeoxycholic acids by the reaction with methylamine, isopropylamine, diethylamine, diisopropylamine or cyclohexylamine in the presence of HOBT or DCC or BOP or methylchloroformate and subsequent oxidation with aluminum tri-*tert*-butoxide or aluminum triisopropoxide or Ag_2_CO_3_ in benzene or toluene or cyclohexane or trifluorotoluene, compounds of type 85 were received, then their reductive amination with amines in the presence of titanium isopropylate produced a series of steroids 86 (Scheme 12) [69]. The biological activity data are presented in Section 6.

### 3.4. Synthesis from 22-Hydroxy-23,24-Dinorchol-4-En-3-One and Its Analogs

**Scheme 13:**
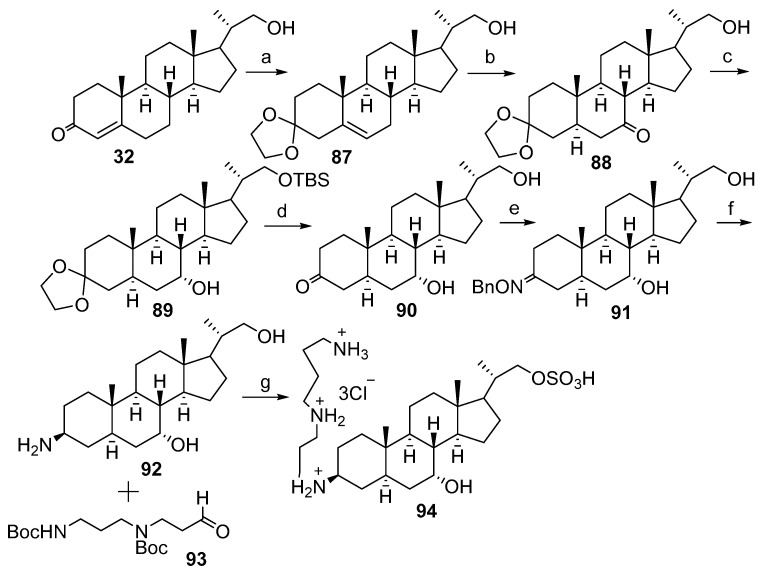
Reagents and conditions: (**a**) 1. OH(CH_2_)_2_OH, PTSA, benzene; 2. Imidazole, TBDMSCl, DMAP, CH_2_Cl_2_; (**b**) 1. RuCl_3_, TBHP, cyclohexane; 2. H_2_, 5% Pt/C, EtOAc; (**c**) K-selectride, THF; (**d**) 1N HCl, THF; (**e**) BnONH_2_ HCl, Py, EtOH; (**f**) LiAlH_4_, Et_2_O; (**g**) 1. NaBH(OAc)_3_, CH_2_Cl_2_; 2. 10% HCl, MeOH; 3. SO_3_-Py, MeOH.

The synthesis of a squalamine analog with a shorter side chain was described in [70]. Starting from 22-hydroxy-23,24-dinorchol-4-en-22-ol 32 by successive transformations, including isomerization of the double bond at position C5 (compound **87**), allylic oxidation to C7 ketone 88, and its stereoselective reduction to 7α-hydroxy-derivative 89, removal of the protective group to form ketone 90, the action of lithium aluminum hydride on 3-benzyloxime 91 3β-amine 92 was obtained (Scheme 13). The reductive amination of 3β-aminosterol 92 with *tert*-butyl *N*-(4-aminobutyl)-*N*-3-(oxopropyl) dicarbonate 93 followed by deprotection and regioselective sulfation formed a conjugate with spermidine in the form of trichlorohydrate 94 [71].

**Scheme 14:**
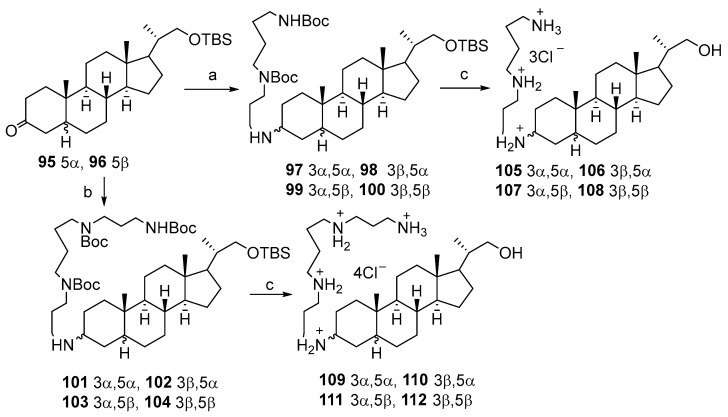
Reagents and conditions: (**a**) NH_2_(CH_2_)_4_NBoc(CH_2_)_3_NHBoc, NaBH(OAc)_3_, THF; (**b**) NH_2_(CH_2_)_3_NBoc(CH_2_)_4_NBoc(CH_2_)_3_NHBoc, NaBH(OAc)_3_, THF; (**c**) SOCl_2_-MeOH, CH_2_Cl_2_.

Steroid ketones 95 and 96 reacted with Boc-substituted spermidine and spermine to form 3α- and 3β-aminobisnorsteroids 97–100 and 101–104, respectively. When they were treated with thionyl chloride, the protecting groups were removed and the hydrochlorides of steroid conjugates 105–112 were synthesized (Scheme 14) [72]. The data concerning their biological activity are presented in Section 6.

### 3.5. Synthesis of Steroid Methylenepolyamines from Cholestan, 4-Cholestene, 5-Cholesten-3-One, 6-Ketocholestanol and 3,7-Diketocholestene

**Scheme 15:**
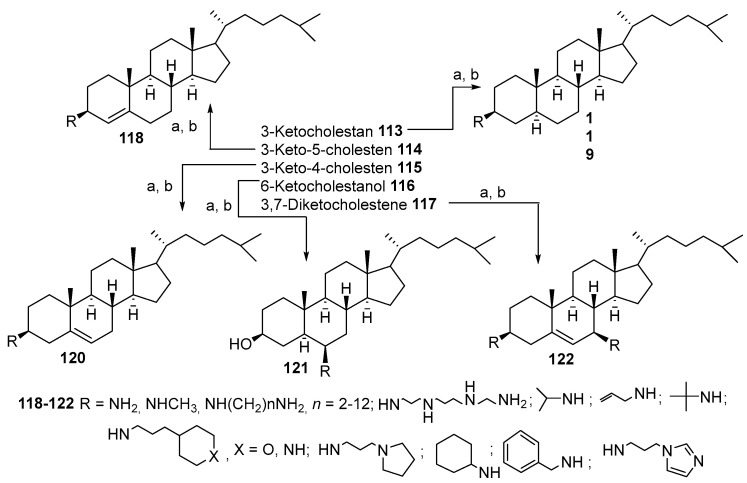
Reagents and conditions: (**a**) RNH_2_, Ti(O*i*Pr)_4_, MeOH, 20 °C, 12 h; (**b**) NaBH_4_, MeOH, −78 °C, 2 h.

The synthesis of more than 30 steroid polyamines with antibacterial activity was described in [73,74,75,76]. Reductive amination of 3-ketones of cholestane 113, 5-cholestene 114, 4-cholestene 115, 6-ketocholestanol 116 and 3,7-diketocholestene 117 with various amines (aliphatic, cyclic, and piperazines) in the presence of Ti(O*i*Pr)_4_ and NaBH_4_ in 41–98% yield led to derivatives of type 118–120 and 121 (Scheme 15). The data concerning their biological activity are presented in Section 6 [73,76].

### 3.6. Synthesis from 3-Keto-7-Hydroxycholestane

**Scheme 16:**
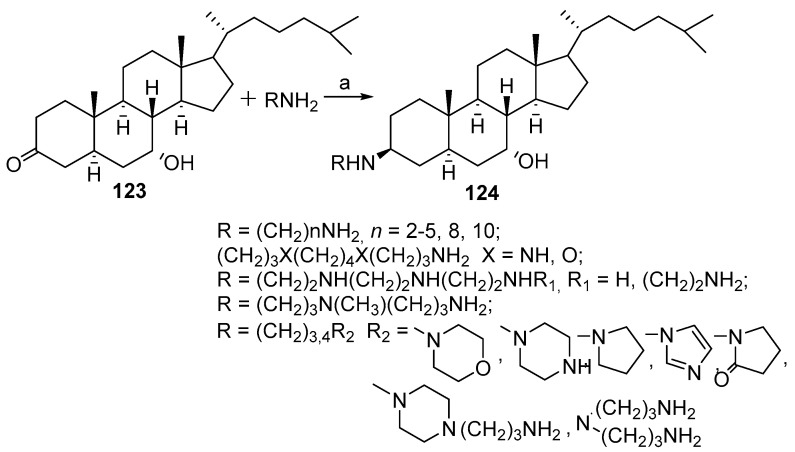
Reagents and conditions: (**a**) 1. RNH_2_, Ti(O*i*Pr)_4_, MeOH, 20 °C, 12 h; 2. NaBH_4_, MeOH, −78 °C, 2 h.

The reaction of reductive amination of 3-keto-7-hydroxycholestane 123 with amines in the presence of titanium isopropylate produced compounds of type 124 (Scheme 16) [69]. The data concerning their biological activity are presented in Section 6 [69].

### 3.7. Synthesis of Squalamine Analogs from Cholesterol and Progesterone

**Scheme 17:**
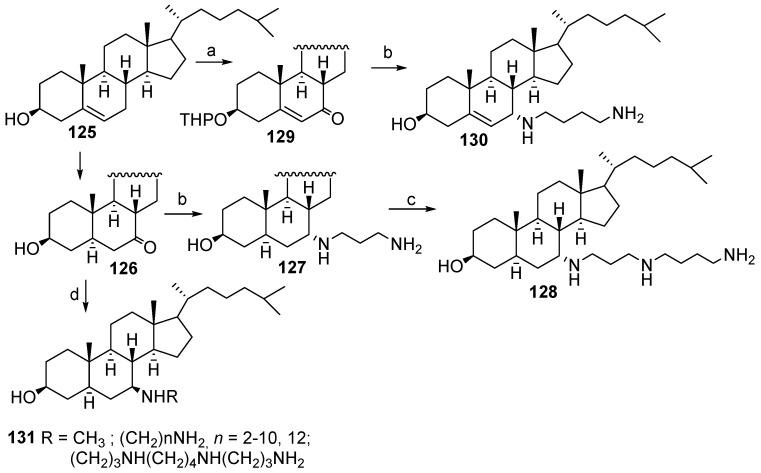
Reagents and conditions: (**a**) 1. Dihydropyran, TsOH, CH_2_Cl_2_, 1.5 h; 2. CrO_3_-Py, CH_2_Cl_2_; (**b**) 1. NH_2_(CH_2_)_3_NHBoc or NH_2_(CH_2_)_4_NHBoc, NaBH_3_CN, AcOH pH 5–6; 2. CF_3_COOH, CH_2_Cl_2_; (**c**) 1. Br(CH_2_)_3_CN, DMF, 60 °C; 2. LiAlH_4_, NiCl_2_ 6H_2_O, THF; (**d**) 1. Amine, Ti(O*i*Pr)_4_, MeOH, 20 °C, 5–6 h; 2. NaBH_4_, −78 °C, 2 h.

The following approaches were used to obtain squalamine analogs containing a polyamine chain at the C7 position. By means of known methods, 3β-hydroxy-7-ketone 126 was obtained from cholesterol 125, the reductive amination of which with diaminopropane, due to steric factors, led to a single 7α-epimer 127 (Scheme 17). Its alkylation with 4-bromobutyronitrile and reduction produced 7α-spermidine-cholesterol 128 [37]. 7α-(1,4-Diaminobutane)-cholest-5-en-3β-ol 130 and 7β-derivatives 131 were synthesized by the reductive amination of ketones 129 or 126 with various amines [73,77]. The data concerning their biological activity are presented in Section 6 [78,79,80].

**Scheme 18:**
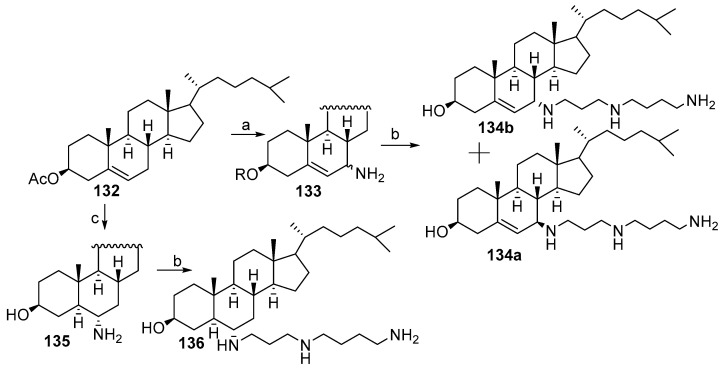
Reagents and conditions: (**a**) 1. (AcO)_4_Pb, (CH_3_)_3_SiN_3_, CH_2_Cl_2_; 2. LiAlH_4_, THF, 66 °C; (**b**) 1. CH_2_CHCN, MeOH; 2. LiAlH_4_,/NiCl_2_ 6H_2_O, THF, 66 °C; 3. Br(CH_2_)_3_CN, DMF, 60 °C, 72 h; 4. LiAlH_4_,/NiCl_2_ 6H_2_O, THF, 66 °C; (**c**) 1. AcOH, HNO_3_; 2. AcOH/Zn; 3. NH_2_OH HCl, Py.

Extension of the polyamine chain at the 7-amino group of compound **133** obtained from 3-acetylcholesterol using cyanoethylation and alkylation with bromobutyronitrile followed by reduction afforded 7α- and 7β-spermidine conjugates 134a and 134b (Scheme 18). The gradual chain extension was performed from 6α-amino derivative of cholesterol 135, obtained in several stages from 3-acetyl-cholesterol 132 (Scheme 18) [37]. As a result of the reactions of its cyanoethylation, a reduction of LiAlH_4_, 6α-spermidinecholesterol 136, was obtained.

**Scheme 19:**
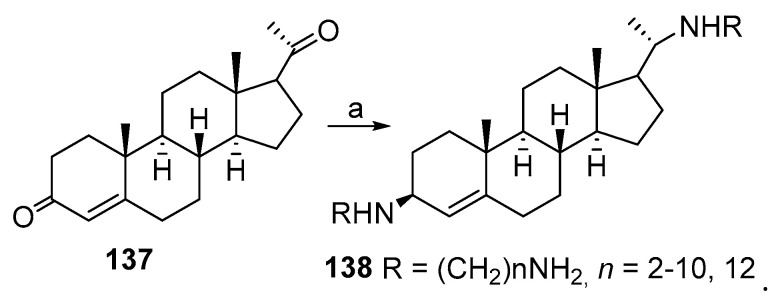
Reagents and conditions: (**a**) 1. RNH_2_, Ti(O*i*Pr)_4_, MeOH, 20 °C, 12 h; 2. NaBH_4_, −78 °C, 2 h.

3,20-Diamino- and polyaminosteroid analogs of squalamine type 138 were synthesized by the reaction of reductive amination of progesterone 137 with various amines in 18–82% yields (Scheme 19). The data concerning their biological activity are presented in Section 6 [77,81,82].

### 3.8. Synthesis of Spermidino-7-Fluoro-3-Aminosteroids

**Scheme 20:**
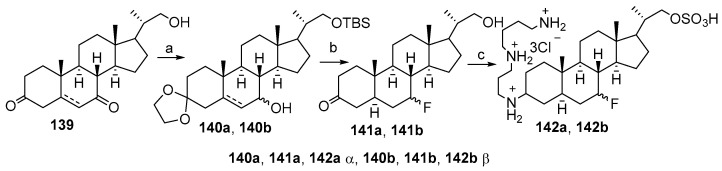
Reagents and conditions: (**a**) 1. HOCH_2_CH_2_OH, PTSA, benzene; TBSCl, imidazole, DMAP, CH_2_Cl_2_; 2. Li/NH_3_, THF; 3. LiAlH_4_, THF; (**b**) DAST, *n*-C_5_H_12_; 2. p-TsOH, acetone; (**c**) 1. NH_2_(CH_2_)_4_NBoc(CH_2_)_3_NHBoc, NaBH_3_CN, THF-MeOH; 2. SO_3_-Py, Py; 3. SOCl_2_, MeOH, CH_2_Cl_2_.

Starting from commercially available 23,24-bisnorchol-4-ene 139, the synthesis of 7β-OH 140b (95%) and 7α-OH 140a (5%) derivatives was carried out in several stages, which included allylic oxidation to ketone, adding dioxolan and *tert*-butylsilyl protection and the action of lithium in liquid ammonia (Scheme 20). Fluorination and subsequent removal of the protective groups led to 7α-141a and 7β-fluoro-23,24-bisnorcholanates 141b in ratio 4:3 with 83% yield [83]. Subsequent reductive amination with Boc-spermidine, sulfation, and removal of Boc-protection led to the target 7-fluoro derivatives 142a, b.

### 3.9. Synthesis of Cholanic Acid Carboxamides with Alkane Polyamines

**Scheme 21:**
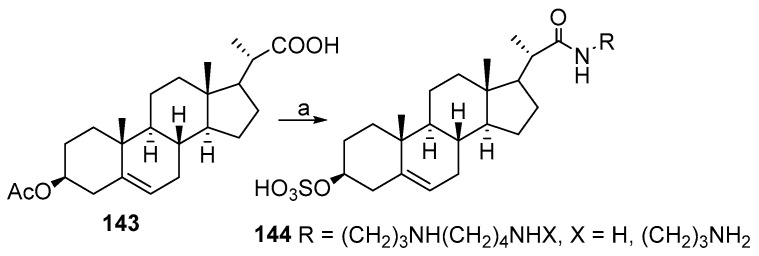
Reagents and conditions: (**a**) 1. NH_2_(CH_2_)_4_NH(CH_2_)_3_NH_2_, NH_2_(CH_2_)_3_NH(CH_2_)_4_NH(CH_2_)_3_NH_2_, DCC, Et_3_N, 7 h, 25 °C; 2. LiAlH_4_, THF, 6 h; 3. SO_3_-Py, Py, 5 h, 75 °C; 4. CF_3_COOH, CH_2_Cl_2_, 1 h, 25 °C.

The interaction of 3β-acetoxy-23,24-dinor-5-cholenic acid 143 with Boc-spermidine or spermine by the DCC method led to amides, characterized as 3β-sulfates 144 (Scheme 21) [84].

**Scheme 22:**
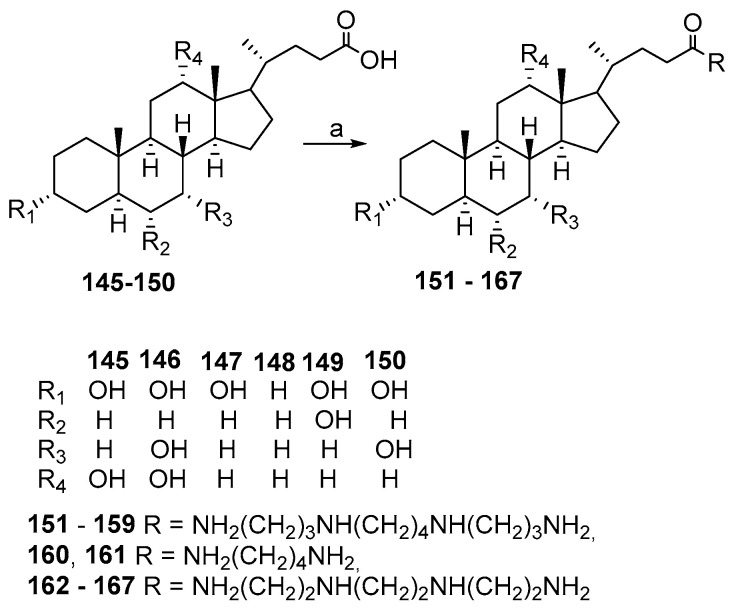
Reagents and conditions: (**a**) 1. DCC, NH_2_(CH_2_)_3_NH(CH_2_)_4_NH(CH_2_)_3_NH_2_, NH_2_(CH_2_)_4_NH_2_, NH_2_(CH_2_)_2_NH(CH_2_)_2_NH(CH_2_)_2_NH(CH_2_)_2_NH_2_, CHCl_3_, 14 h, 25 °C; 2. SO_3_-Py, DMF.

Interaction of 3α,12α-dihydroxycholanic 145, 3α,7α,12α-trihydroxycholanic 146, 3α-hydroxycholanic 147, ursocholanic 148, 3α,6α-dihydroxycholanic 149, 3α,7α-dihydroxycholanic 150, and 23,24-bisnor-5-cholenic 151 acids with spermidine, triethylenetetramine or putrescine in the presence of DCC, followed by a deprotection afforded amides 151–167 (Scheme 22) [9,85,86]. The data concerning their biological activity are presented in Section 6 [50,56].

**Scheme 23:**
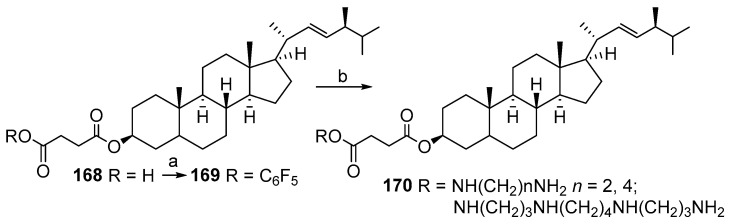
Reagents and conditions: (**a**) bis(pentafluorophenyl)carbonate, 4-methylformalin, DMF, 1.5 h; (**b**) NH_2_(CH_2_)_4_NHBoc (*n* = 2, 4), NH_2_(CH_2_)_3_NBoc(CH_2_)_4_NBoc(CH_2_)_3_NHBoc, C_8_H_19_N, DMF, 2 days.

Stigmasterol succinate 168 was activated by the formation of pentafluorophenyl succinate 169, after removal of the protective group and interaction with Boc-polyamines, followed by removal of the Boc-protection, carbamates of type 170 have been synthesized (Scheme 23). The data concerning their biological activity are presented in Section 6 [87].

**Scheme 24:**
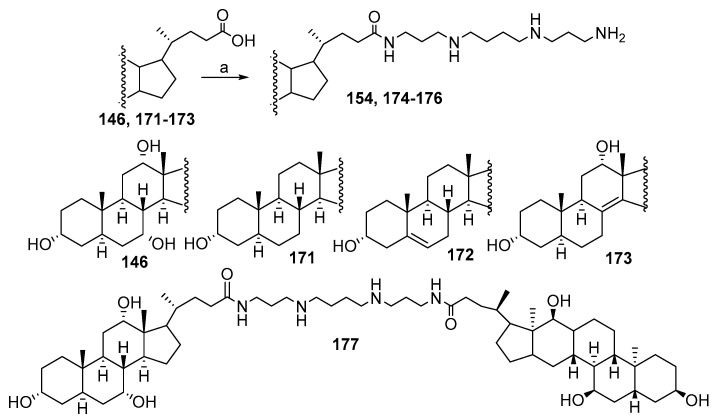
Reagents and conditions: (**a**) NH_2_(CH_2_)_3_NH(CH_2_)_4_NH(CH_2_)_3_NH_2_, DCC, BuOH, THF.

Compounds 156, 171–177 were synthesized by conjugation of spermine with cholic acids 146, 171–173 (Scheme 24). The data concerning their biological activity are presented in Section 6 [88].

### 3.10. Synthesis of Steroid Carbamates

**Figure 2:**
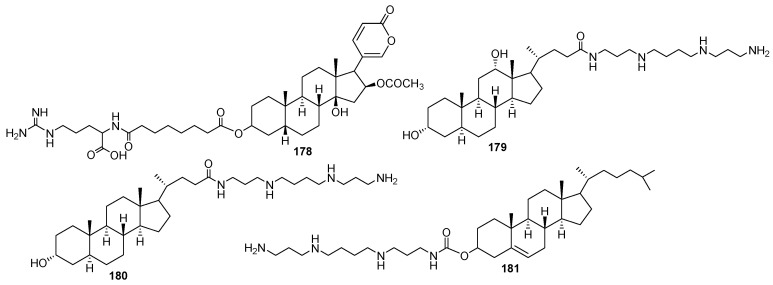
The structures of compounds 178–181.

A representative example of natural polyamine steroidal carbamates is bufotoxin 178, isolated from the venom of the toad *Bufo Vulgaris*, which disrupts the work of the heart muscle (Figure 2) [86]. Synthetic steroidal lipopolyamines 179–181 are more efficient than natural polyamines spermine and spermidine. Similar compounds can be used in fluorescence correlation spectroscopy as a means of studying supramolecular formations in gene delivery systems, and in non-viral gene therapy [89].

**Scheme 25:**
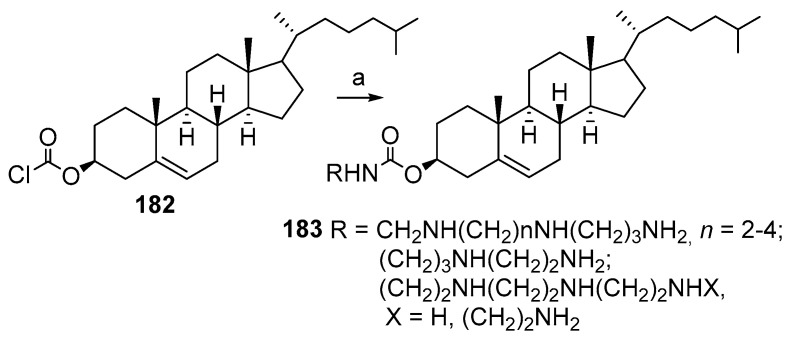
Reagents and conditions: (**a**) RNH_2_, CH_2_Cl_2_, Et_3_N, 0 °C, 10 min/25 °C, 12 h.

Synthetic approaches to the preparation of steroidal polyamine carbamates included the introduction of polyamine moieties at the C3 position or to the side chain. For example, the interaction of 3-cholesteryl chloroformate 182 with polyamines (spermine, 1,11-diamino-4,8-diazaundecane, 1,10-diamino-4,7-diazadecane, 1,9-diamino-3,7-diazanonane, tetraethylenepentamine, pentaethylenehexamine) led to 3-cholesterylpolyamine carbamates of type 183, which can be considered as a model for the formation of lipoplex (complexes of cationic, neutral lipids and DNA molecules, used as a system for cell transfection), which are key steps in gene therapy (Scheme 25) [90].

**Figure 3:**
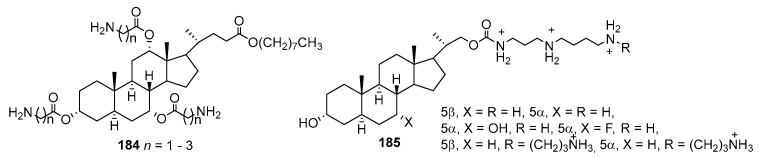
The structures of compounds 184, 185.

Carbamates 184 were useful in inhibiting the growth of bacteria in food due to the manifestation of weak basic properties in the gastrointestinal tract [35]. Carbamates 185, derivatives of cholic acid with Boc-spermidine or Boc-spermine (Figure 3) were tested against Gram-positive and Gram-negative bacteria [35,91,92].

### 3.11. Synthesis of Aminopropoxysteroids

**Figure 4:**
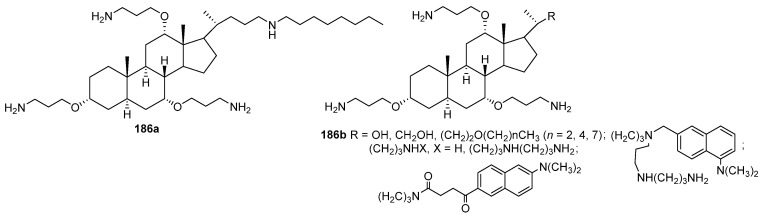
The structure of ceragenins 186a and 186b [60,103].

3,7,12-*tris*-Aminopropoxysteroids with various side-chain substituents, named as ceragenins, were synthesized to mimic cationic peptide antibiotics such as polymyxin B [33,93]. Ceragenins have a broad spectrum of antibacterial activity. Ceragenin 186a (Figure 4) is active against *H. pylori* and against cariogenic and periodontopathic bacteria with MICs 0.275–8.9 and 1–16 μg/mL, respectively [93,94,95], was recommended for the treatment of chronic infections and inflammation in patients with cystic fibrosis [95,96,97], including for local application [98]. A technology was proposed that combines the antibacterial effect and medical imaging of ceragenin 186a with magnetic nanoparticles [41,99,100]. More details on its activity are presented in Section 6 [38,101,102,103]. The introduction of an aminopropoxy group into the cholic acid scaffold included alkylation with allylbromide followed by a reduction of the azido group [50]. Ceragenins 186b with various substituents in the side chain possessing antibacterial activity have been also synthesized [50,104].

### 3.12. Synthesis of Squalamine Phosphate

**Figure 5:**
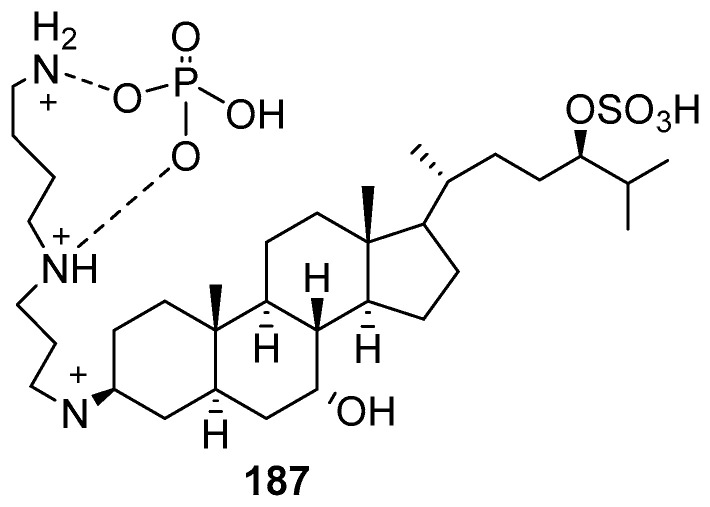
The structure of squalamine phosphate 187.

Zasloff et al. has synthesized aminosterol phosphate compositions and discovered their biological activity as anti-inflammatory, anti-viral, antimicrobial, and antifungal agents. The aminosterol phosphate compositions permit administration without associated tissue damage and achieve a sustained-release effect. Squalamine phosphate 187 (Figure 5) can be prepared simply by adding a soluble phosphate salt (i.e., sodium, potassium, ammonium) to a solution of squalamine [105].

### 3.13. Synthesis of Squalamine Analogues with Multiple Steroid Backbones

**Scheme 26:**
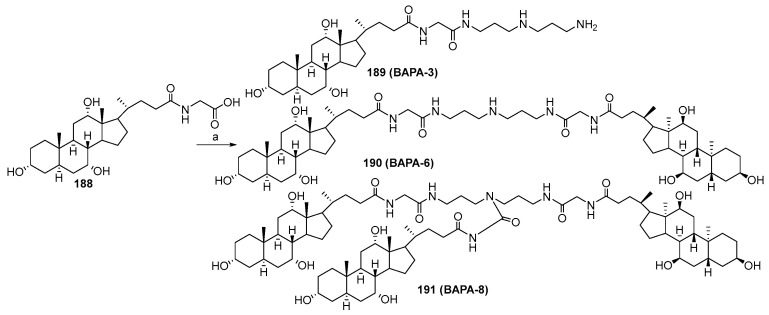
Reagents and conditions: (**a**) NH_2_(CH_2_)_3_NH(CH_2_)_3_NH_2_, EEDQ, DMF, 60 °C, 4 h.

Derivatives 189–191 were synthesized on the basis of cholylglycine, containing one, two, or three steroid scaffolds linked through *N*-(3-aminopropyl)-1,3-propanediamine as a spacer in 8.5%, 6%, and 2% yields, respectively (Scheme 26) [106]. The data concerning their biological activity are presented in Section 6 [106].

**Scheme 27:**
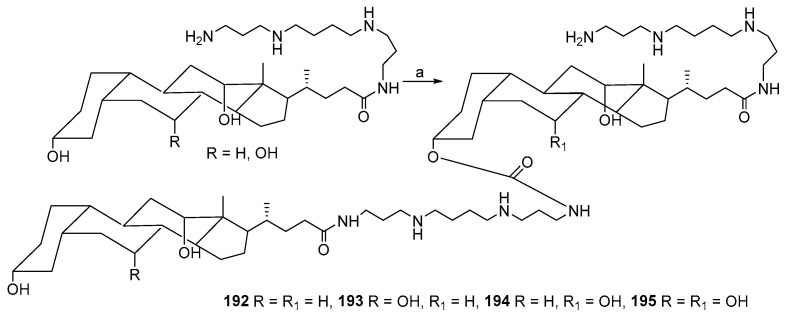
Reagents and conditions: (**a**) DSC, Et_3_N, CHCl_3_, acetonitrile, 50 °C, 3 h.

The synthesis of dimeric conjugates 192–195 of cholic and deoxycholic acids (Scheme 27), which consist of two amphiphilic sterol-spermine units linked to each other by a carbamate moiety in the form of a head-tail, using DSC has been reported [107]. The data of biological activity are presented in Section 6 [108]. Conjugate 195 exhibited the same activity as squalamine, suggesting its use as a potential antibacterial agent [109].

**Scheme 28:**
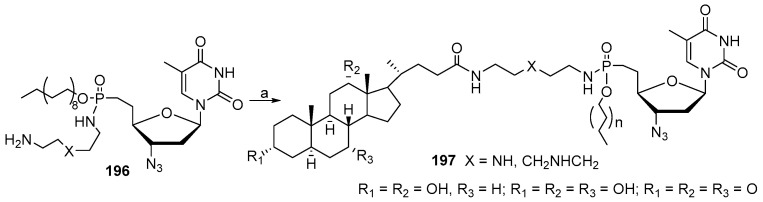
Reagents and conditions: (**a**) Succinimidyl ester of deoxycholic acid, CHCl_3_, r.t.

The synthesis of phosphoramide conjugates of bile acids with 3′-azido-3′-deoxythymidine was described in [110,111,112]. The reaction of the polyamine derivative of phosphoroamide 196 with the acylchlorides of deoxycholic, cholic, and dihydrocholic acids led to polyaminoazidothymidine conjugates 197 (Scheme 28) [113]. The data of biological activity are presented in Section 6 [113].

**Scheme 29:**
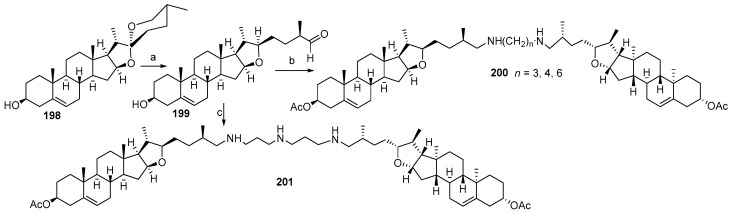
Reagents and conditions: (**a**) 1. Ac_2_O, Et_3_N, DMAP, CH_2_Cl_2_, 24 h; 2. NaBH_3_CN, AcOH, 48 h; 3. PCC, CaCO_3_, SiO_2_, CH_2_Cl_2_, 12 h; (**b**) 1. 1 eq. 4 DCE, AcOH, 1 eq diamine; 2. AcOH, NaBH(OAc)_3_, 4 days. (**c**) 1. 1 eq 4 in DCE, AcOH, 0.5 eq spermidine; 2. AcOH, NaBH(OAc)_3_, 4 days.

Shawakfeh et al. has synthesized dimeric derivatives based on diosgenin 198. The dimers were formed through the amination reaction with 1,3-diaminopropane, 1,4-diaminobutane, 1,6-diaminohexane, and spermidine and the aldehyde group of steroid 199 obtained as a result of the F-ring opening of diosgenin acetate in high yields (Scheme 29) [114].

**Figure 6:**
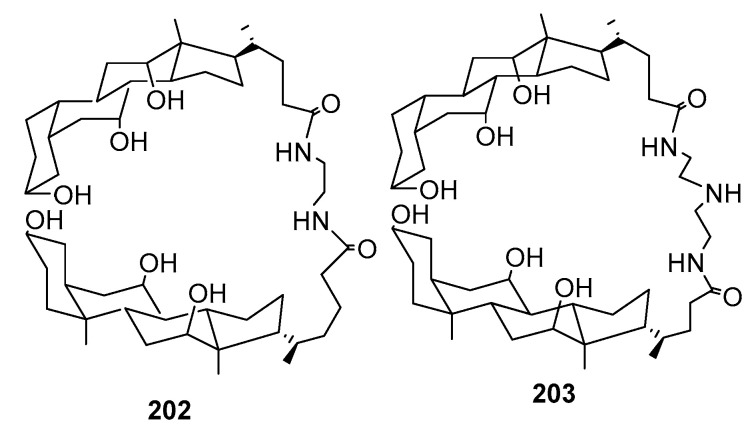
The structures of compounds **202**, **203**.

Compounds with two steroid scaffolds 202 and 203 (Figure 6) possess antifungal activity against five types of fungi and against cancer cells HEp-2 and MCF-7 [36].

**Figure 7:**
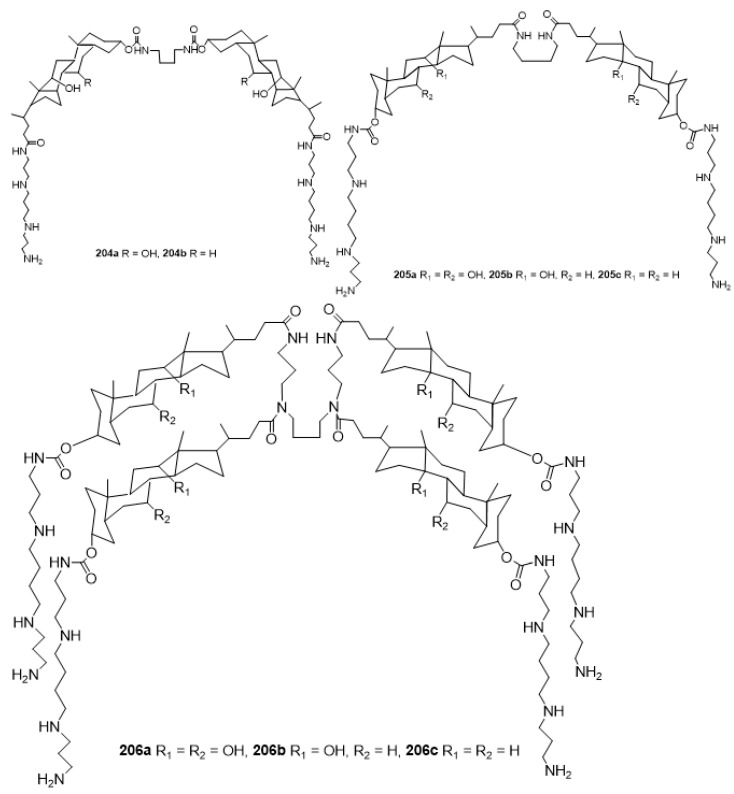
The structures of compounds **204**–**206**.

Umbrella dimers 204a,b, 205a–c, and tetramers 206a–c (Figure 7) are more active than monomeric analogs of squalamine, the presence of hydroxyl groups at C7 and C12 leads to high activity compared to analogs with a hydroxyl group only at C12. The results indicate that such conjugates act as antibiotics at the membrane level through the pore and channel formation. Compound 204a is responsible for the activation of pH bischarging across liposomal membranes at the level of antibiotic activity, which is comparable to the monomeric analogs of squalamine. Monomeric and dimeric analogs of squalamine are present in the bacterial membrane in an inactive form, and only small fractions are in the form of clusters that activate ion transport. Head-to-tail dimers are more active than head-to-head dimers [108].

Dimeric and tetrameric analogs in which two or four subunits were linked by a side chain to putrescine or spermine “head-to-head” or “tail-to-tail” demonstrated high antibacterial activity [108], Chen et al. became interested in conducting a detailed study structures-activity using linked sterol-polyamine conjugates, i.e., covalently linked dimers and tetramers. They synthesized a number of dimeric and tetrameric analogs, in which two or four subunits were linked a head-to-head or tail-to-tail to putrescine or spermine backbone [108].

Studies of antibacterial activity have shown that dimeric conjugates exhibit strong antibacterial activity against a wide range of gram-positive bacteria, while tetrameric conjugates exhibit very weak properties. The latter is believed to be a likely consequence of either an unfavorable steric interaction with peripheral proteins, or the result of a relatively high water solubility, which may prevent their efficient separation in the plasma membrane, or both of these factors. Dimeric and tetrameric conjugates of lithocholic acid did not show antibacterial activity. The lack of activity may be due to the lack of amphiphilicity.

**Scheme 30:**
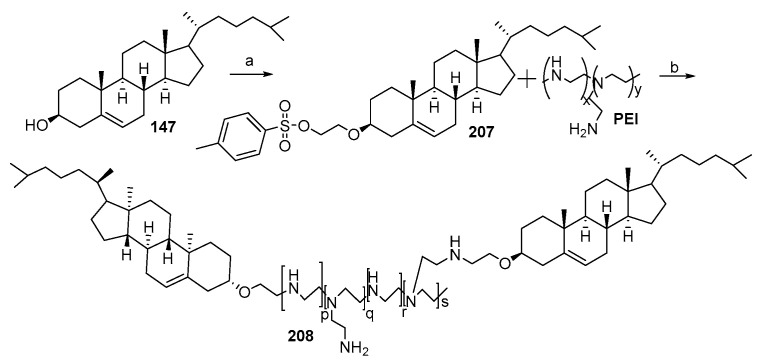
Reagents and conditions: (**a**) 1. p-TsCl/Py/CHCl_3_, 0 °C, 6 h; 2. HO(CH_2_)_2_OH, 1,4-dioxane, reflux, 4 h; 3. p-TsCl/Py/CHCl_3_, 0 °C, 6 h; (**b**) MeOH, toluene, reflux.

Cholest-5-en-3β-oxyethane-tosylate 207 was synthesized using known approaches such as tosylation, saponification with ethylene glycol, and repeated tosylation. Further to a solution of cholest-5-en-3β-oxyethane-tosylate 207 in dry toluene desired amounts of PEI in dry MeOH was added, the reaction mixture was refluxed to produce lipopolymers type 208 (Scheme 30) [115]. These compounds have high transfection properties, low cytotoxicity, and high serum compatibilities. The transfection efficacies and cytotoxicity of the lipopolymers were found to be dependent on the percentage of cholesterol grafting and the molecular weight of PEI used for the synthesis of lipopolymers [116].

## 4. Synthesis of Trodusquemine and Its Analogs

**Scheme 31:**
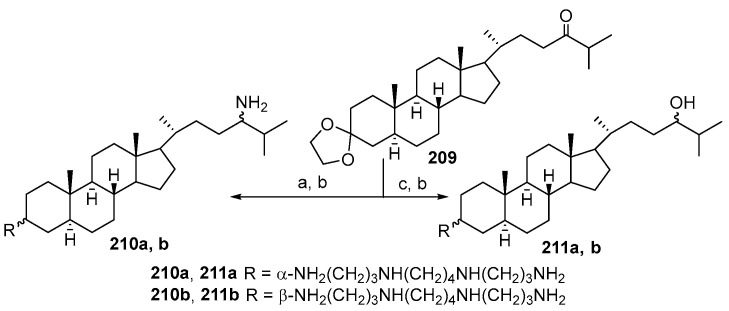
Reagents and conditions: (**a**) NaBH_3_CN, NH_4_OAc, MeOH; (**b**) 1. HCl, acetone; 2. NH_2_(CH_2_)_3_NH(CH_2_)_4_NH(CH_2_)_3_NH_2_, NaBH_3_CN, MeOH; (**c**) NaBH_4_, MeOH, 96%.

The synthesis of trodusquemine 2, which is a spermine analog of squalamine 1, is also based on a reductive amination reaction [34]. Conjugates of 24-amino- and 24-hydroxy 3α- and 3β-cholestane derivatives with spermine 210a–b, 211a–b were obtained by reductive amination from 3,3-(ethylenedioxy)-cholestan-24-one 209 (Scheme 31) [34].

**Scheme 32:**
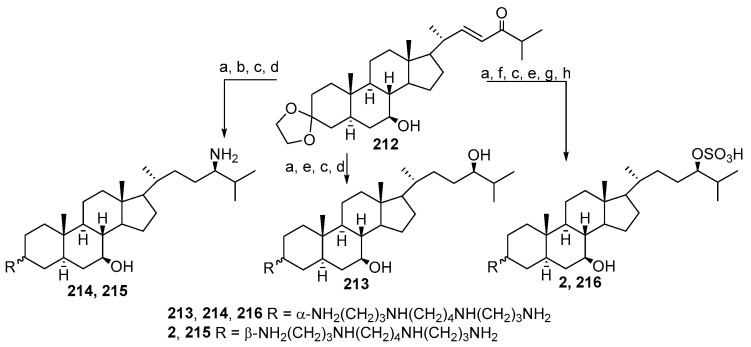
Reagents and conditions: (**a**) H_2_, Pd/C, MeOH, 90%; (**b**) NaBH_3_CN, NH_4_OAc, MeOH, 72%; (**c**) HCl, acetone, 68%; (**d**) NH(CH_2_)_3_NH(CH_2_)_4_NH(CH_2_)_3_NH_2_, NaBH_3_CN, 81%; € NaBH_4_, MeOH, 87%; (**f**) BnONH_2_ HCl, DMAP, Py, 90%; (**g**) SO_3_-Py, Py, 84%; (**h**) NH_2_(CH_2_)_3_NH(CH_2_)_4_NH_2_, NaBH_3_CN, LiOH, 76%.

According to a similar approach, conjugates of 24-amino-, 24-hydroxy- and 24-sulfate-3α- and 3β,7β-hydroxy-5α-cholestane derivatives 2, 213–216 with spermine were synthesized from 3,3-(ethylenedioxy)-7β-hydroxy-5α-cholest-22-ene 212 (Scheme 32) [34]. The data of the biological activity of this series of compounds is presented in Section 6 [117].

## 5. Synthesis of Claramine and Its Analogues

**Scheme 33:**
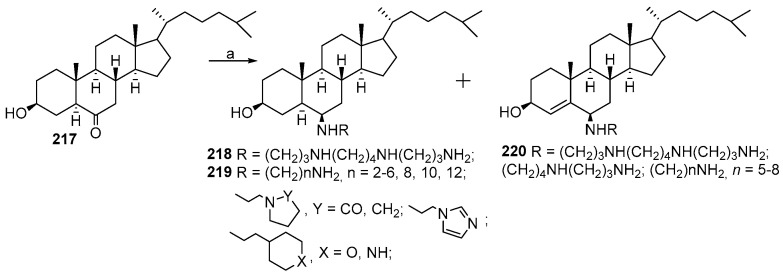
Reagents and conditions: (**a**) 1. RNH_2_, Ti(O*i*Pr)_4_, MeOH, 20 °C, 12 h; 2. NaBH_4_, MeOH, −78 °C, 2 h.

Chen et al. and Govers et al. obtained an analog of trodusquemine-claramine 218, which is a conjugate of 3β-hydroxy-6β-cholestan with spermine. Claramines 219 and 220 were also synthesized by reductive amination reaction (Scheme 33) [118]. The data of biological testing is presented in Section 6 [119].

**Scheme 34:**
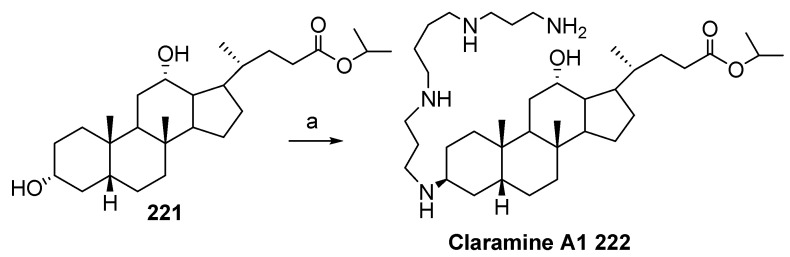
Reagents and conditions: (**a**) 1. R_1_OH, PTSA, CH_2_Cl_2_, 60 °C, 8 h; 2. Al(OBu)_3_, acetone, toluene, 110 °C; 3. NH(CH_2_)_3_NH(CH_2_)_4_NH(CH_2_)_3_NH_2_, Ti(O*i*Pr)_4_, MeOH, 20 °C, 24 h; 4. NaBH_4_, −78 °C.

Starting from deoxycholic acid derivative 221 Blanchet et al. has obtained claramine A1 222 in three stages by reductive amination with spermine with a total yield of 33% (Scheme 34). The data of activity could be seen in Section 6 [120].

Summarizing the above results, we can conclude that the approach to the synthesis of analogs of squalamine, trodusquemine, and claramine was based on the introduction of a polyamine fragment at positions C3, C6, C7, C12, and C24 of the steroid scaffold by the reactions of reductive amination, cyanoethylation of amines, and acylation (synthesis of derivatives with two and three steroid fragments, steroid analogs of polymyxin B).

## 6. Biological Properties of Squalamine, Trodusquemine and Their Analogues

The diverse biological activity of aminosterols has been subjected to numerous reviews. Previous works covered the activity, mechanism of action, and prospects of squalamine and similar aminosterols as a new class of antibiotics capable of overcoming the problem of resistance [11,33,38,42,80,121], as well as their antifungal and antiviral properties [47,48]. Special attention was paid to their anti-angiogenic activity for the treatment of tumor diseases [122]. Antibacterial, fungicidal, and immunomodulatory properties of ceragenins and ceragenin-derived nanoparticles were recently reviewed in [123,124].

### 6.1. Biological Activity of Squalamine and Trodusquemine

#### 6.1.1. Antibacterial and Antiviral Activity

Squalamine initially became known as a broad-spectrum bactericidal antibiotic effective against both Gram-positive and Gram-negative bacteria, including *E. coli*, *P. aeruginosa*, *S. aureus*, *S. faecalis*, *P. vulgaris* [38]. Later, squalamine was found in the membrane of sea lamprey (*P. marinus*) leukocytes [10], which confirmed its role as an important factor of humoral immunity [125]. The different electric charge of prokaryotic and eukaryotic cells allows squalamine to selectively bind to bacterial membranes [126], exhibiting low minimum inhibitory concentrations (MIC 1–8 μg/mL). At the same time, the minimum concentration causing hemolysis of erythrocytes exceeds 200 μg/mL, and it is not genotoxic [127]. Furthermore, unlike beta-lactam antibiotics, which have a similar spectrum of antibacterial activity, squalamine is a fungicide (*C. albicans*, *A. fumigatus*, *A. niger*, *Fusarium* spp.) and causes osmotic lysis of protozoa (*P. caudatum*).

It is especially important that squalamine retains activity even against clinically important multi-resistant strains of *E. coli* and *P. aeruginosa*, overexpressing various factors of resistance, including active excretion of drugs, changes in membrane permeability caused by the absence of porins, an enzymatic barrier that induces resistance to quinolones, β-lactam, phenicols, etc. [128]. In particular, it effectively eradicates fungi and multi-resistant Gram-negative and Gram-positive bacteria isolated from patients with cystic fibrosis [78,79] and fungemia [129]. Squalamine and its analog 138 are active against mupirocin-susceptible and resistant clinical isolates of *S. aureus* with MIC values of 3.125 µg/mL. Additionally, repeated exposure of a *S. aureus* strain to squalamine and 138 did not lead to the emergence of resistant bacteria, contrarily to mupirocin [82]. Trodusquemine 2 has a broad spectrum of antimicrobial activity (MIC 1–4 μg/mL for *S. aureus*, *P. aeruginosa*, and *C. albicans*), slightly outperforming squalamine [7].

In addition, squalamine at a concentration of 0.5–1 μg/mL causes the death of archaea species, e.g., *M. smithii*, *M. oralis*, *M. arboriphilicus*, *M. concilii*, and *M. beijingense* [130,131], and can be used to disinfect medical instruments instead of aggressive peracetic acid. At a concentration of 100 µg/mL, squalamine effectively destroys dormant cells of the causative agent of nosocomial infections *A. baumannii*, which are resistant to ciprofloxacin therapy [132]. Squalamine showed significant in vitro activity against *Trichophyton* and *Microsporum* dermatophytes with MICs ranging from 4–16 μg/mL (1–4 μg/mL for griseofulvin) [133].

Squalamine is a membrane-active compound. The bactericidal activity of squalamine is attributed to the combination of anionic bile acid with cationic spermidine, which individually exhibits significantly lower antibiotic activity [134,135]. The mechanism of action of squalamine is similar to cationic peptide antibiotics and consists of a selective violation of the integrity of the bacterial membrane or the formation of semi-stable pores in it due to electrostatic binding with phospholipids, followed by depolarization [126,136]. The selectivity of squalamine is explained by its affinity for bacterial lipopolysaccharides and the ability to penetrate into the lipid bilayer [137]. Disruption of the barrier function of the bacterial membrane leads to depletion of the intracellular ATP pool (loss of 80% ATP at a concentration of 20 μg/mL) and cell death [128]. Squalamine has the highest affinity for phosphatidylglycerol (the main component of bacterial membranes), and somewhat less for phosphatidylserine and cardiolipin [31]. Using fluorescently labeled dextrans, it was found that squalamine increases membrane permeability for substances with molecular weights up to 4 kDa, but less than 10 kDa. Moreover, its activity is completely suppressed by the presence of 5 mM Ca^2+^ or Mg^2+^ ions [128], which indicates a direct interaction of squalamine with membrane phospholipids. It is not a protonophore [138]. The surface antigen of *E. coli* O4 reduces the effectiveness of squalamine, while K54 has a sensitizing effect. The mechanism remains unclear [139]. Additionally, a recent study demonstrated that squalamine competitively inhibits the glycosyltransferase activity of penicillin-binding proteins of *E. coli*, which mediates the cell wall synthesis, although only in high concentrations (IC_50_ 291 µM) [76].

**Figure 8:**
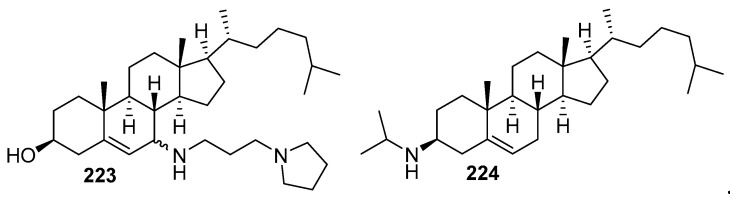
The structures of compounds **223** and **224**.

The specificity of the interaction of squalamine with negatively charged phospholipids is confirmed by its inability to induce the death of mycobacteria, whose cell wall consists of arabinogalactan esterified with residues of fatty mycolic acids. Ghodbane et al. designed squalamine analogs where spermidine was replaced with other alkylamines to increase lipophilicity of compounds 223, 224 (Figure 8), which rendered them active against several mycobacteria species (MIC 5–25 μg/mL), but they did not affect the viability of the tuberculosis causative agent *M. tuberculosis* [140]. Squalamine itself, due to its selectivity towards bacteria, and especially *S. aureus* (MIC 3.12 μg/mL) and *P. aeruginosa* (MIC 8 μg/mL) can be used for decontamination and isolation of mycobacteria from sputum samples [141].

Further studies have shown that the membrane-permeabilizing effect of squalamine potentiates the activity of chloramphenicol, tetracycline, ciprofloxacin, etc. The combined use of squalamine in a subinhibitory concentration with antibiotics makes it possible to reduce their dose and overcome the resistance of antibiotic-resistant strains of *E. aerogenes* ATCC 13048 and CM-64, *P. aeruginosa* PA01 and PA124, *K. pneumoniae* KP63 and KP55, *E. coli* AG100 and AG100a [103].

Squalamine displayed great efficacy against *A. baumannii* dormant cells (i.e., persisters, which are responsible for recurrent infections) at the 100 μg/mL dose (below the minimum hemolytic concentration) [132].

In a mouse model, topical application of squalamine more effectively removes *S. aureus* from the skin than treatment with antiseptic mupirocin used in surgical practice [142]. Squalamine tablets have been developed to disinfect home nebulizers for patients with cystic fibrosis [143]. A squalamine concentration of 0.5 g/L 20 min is enough for disinfection. Aerosol of 3 mg squalamine showed efficacy exceeding 160 mg of colistin in rats with chronic pneumonia caused by *P. aeruginosa* [144]. Treatment with 1% squalamine ointment resulted in clinical improvement in patients with shingles after 3 weeks [145]. On the downside, squalamine is inactivated by calcium and magnesium cations (1 mM of Ca^2+^ blocks the activity of 2.5 μg/mL squalamine, whereas a normal range of Ca^2+^ in human serum is 2.0–2.5 mM [129]), which likely renders it ineffective against systemic infections.

A broad-spectrum antiviral activity has been described for squalamine (Dengue virus, hepatitis B, yellow fever, herpesviruses), indicating that squalamine interaction with cellular membranes prevents the adhesion and fusion of RNA and DNA viruses into cells [146,147]. Due to the positively charged spermidine moiety and affinity for anionic phospholipids, squalamine neutralizes the negative charge of the inner membrane of eukaryotic cells, displacing proteins electrostatically bound to the membrane, in particular Rac1 GTPase used by viruses to enter the cell. At the same time, no disruption or permeabilization of the cell membrane was observed. Zasloff et al. demonstrated a protective effect of parenteral squalamine administration against yellow fever and eastern equine encephalitis in Syrian hamsters and cytomegalovirus infection in BALB/c mice [30].

#### 6.1.2. Neuroprotective Activity

Squalamine prevents aggregation of alpha-synuclein (αS) and competes with it for binding to phospholipid membranes (K_D_ of squalamine 67 nM versus 380 nM for synuclein), which can be used to treat Parkinson’s disease [148]. Recent experiments confirmed that squalamine attenuates the toxicity of αS and amyloid-beta (Aβ) by altering their aggregation and displacing them from cell membranes [149]. Similar properties were later shown for trodusquemine and αS, amyloid-beta (Aβ), and HypF-N oligomers [118,150,151,152]. Squalamine effectively restores disordered colonic motility by restoring excitability of the enteric nervous system in a mouse model [15] and reduced toxicity of αS in a *C. elegans* model of Parkinson’s disease [153]. In experiments modeling Alzheimer’s disease in *C. elegans*, trodusquemine reduced the toxicity of Aβ aggregates by preventing their binding to cell membranes [154]. FRET and NMR studies revealed that polyamine tails of trodusquemine modulate physicochemical properties of the cell membranes themselves, making them more resistant to neurotoxic aggregates of misfolded proteins [118,155]. In a mouse model of Alzheimer’s disease, trodusquemine rescued NMDA-mediated neuronal plasticity [156] and prevented cognitive decline [157]. This highlights the potential of squalamine and trodusquemine for the treatment of Alzheimer’s and Parkinson’s diseases [158].

#### 6.1.3. Antiangiogenic and Antitumor Activity

In 1998 Sills et al. showed that squalamine effectively inhibits angiogenesis and tumor growth in several animal models [14]. The authors linked the suppression of tumor neovascularization with the blocking of mitogen-induced proliferation and migration of endothelial cells. Squalamine has no significant effect on unstimulated endothelial cells and does not have a direct cytotoxic effect on tumor cells, nor does it alter the production of mitogens by tumor cells [159]. One of the components of antiangiogenic action is inhibition of sodium hydrogen exchanger NHE3 of endothelial cells through the C-terminal 76-amino acid fragment [160]. Moreover, it has been shown that squalamine prevents only mitogen-stimulated proliferation and migration of endothelial cells [14]. Besides, squalamine is the first calmodulin chaperone described, causing the translocation of the latter from the cell periphery to perinuclear endosomes [123], which can prevent signal transduction from mitogen receptors. Williams et al. showed that squalamine disrupts actin polymerization and intercellular cadherin-mediated adhesion of endothelial cells [161]. As a result, squalamine prevents mitogen-stimulated activation, migration, coordination, and proliferation of endothelial cells, and thus prevents neovascularization of tumors.

Squalamine itself has only a moderate effect on tumor growth [162]. However, its combination with cyclophosphamide, cisplatin, 5-fluorouracil, and paclitaxel sensitizes the tumor, delaying its growth 1.9–3.8 times compared with monotherapy with cytostatic drugs, which was first shown in rats with breast carcinoma and Lewis lung carcinoma [162]. The effectiveness of combined antitumor therapy with squalamine and platinum drugs has been confirmed by several preclinical studies using lung carcinoma [161]. The combination of squalamine with cisplatin is effective in ovarian cancer, including those with HER-2 overexpression, which is resistant to cisplatin monotherapy [150]. Furthermore, squalamine inhibits the growth of HER-2-negative breast cancer MCF-7 and HER-2-positive MCF-7 in combination with trastuzumab by blocking the action of the endogenous activator of angiogenesis VEGF [163].

It was suggested that squalamine is promising for other diseases characterized by neovascularization. It was shown that squalamine at a dose of 25 mg/kg/day subcutaneously is effective in a model of oxygen-induced retinopathy in mice [164] and suppresses neovascularization after laser injury in rats [165] and macaques even upon systemic administration [166].

### 6.2. Trodusquemine as a Unique PTP1B Inhibitor

#### 6.2.1. Antiobesity and Weight Loss Activity

Further research expanded the known spectrum of biological activity of trodusquemine. It has been shown to inhibit HIV replication in human monocytes [167]. Additional studies carried out on various cell cultures found that the compound also affects the ionic currents of calcium, chloride, and protons [160,168]. In particular, in frog oocytes, trodusquemine caused the calcium-dependent opening of chlorine channels [169]. Surprisingly, it has been found that the administration of trodusquemine induces weight loss in rodents, dogs, and monkeys, which has prompted an in-depth study of the pharmacological properties and mechanism of action of the compound.

Trodusquemine has been shown to induce a reversible decrease in food and fluid intake in mammals, resulting in significant weight loss not associated with side effects, and exhibiting antidiabetic properties in genetically obese mice. Trodusquemine is active when injected into the third ventricle of the rat brain, suggesting a central mechanism of action. When trodusquemine was injected into db/db dyslipidemic mice, a decrease in adipose tissue and a correction of hyperglycemia were noted. Correction of obesity and glucose tolerance was shown in both genetically obese (ob/ob) and diabetic (db/db) mice [170]. The post-receptor mechanism of action of the compound was hypothesized [171].

The study by Ahima et al. confirmed these observations [172]. It was found that the main changes induced by trodusquemine are concentrated in the paraventricular nucleus of the hypothalamus. This area of the brain integrates nerve signals from the nuclei of the hypothalamus and the nucleus of the brain, regulating feeding behavior and several neuroendocrine functions. The introduction of trodusquemine into this region reduced the mRNA levels of the agouti-related peptide and neuropeptide Y in the hypothalamus, suppressing orexigenic pathways.

Many of the drugs that reduce food intake and body weight work in part by blocking the dopamine transporter, a protein responsible for the uptake of extracellular dopamine. Evaluation of the effect of trodusquemine on DAT function did not reveal significant changes in dopamine secretion and degradation while maintaining suppression of food intake [173].

Protein tyrosine phosphatase 1B negatively regulates signaling pathways of leptin and insulin, dephosphorylating their receptors and downstream components of the cascades. The important role of PTP1B in the pathogenesis of obesity and diabetes mellitus was confirmed by the deletion of the PTP1B gene in mice. Mice completely knocked out for the PTP1B gene were protected from the development of obesity and diabetes. Moreover, selective deletion of the PTP1B gene in the brain had the same effect on the weight and carbohydrate metabolism of animals. Deletions in muscle, liver, and adipocytes have no beneficial effect [174,175]. Although these results indicate the importance of PTP1B neuronal activity in maintaining energy homeostasis, peripheral PTP1B is also being investigated as a potential regulator of energy balance. In particular, the important role of hepatic PTP1B expression in glucose homeostasis and endoplasmic stress has been shown [176,177]. PTP1B activity is increased in obesity and type 2 diabetes and is a major cause of insulin resistance. The validation of PTP1B as a therapeutic target for obesity and diabetes has given rise to the development of selective inhibitors of PTP1B [178,179]. These efforts have led to the discovery of several classes of inhibitors, but their therapeutic potential has long been limited by low oral bioavailability [180].

As noted above, trodusquemine induces rapid and reversible weight loss in genetic models of obesity. To better understand the potential effects in the clinic, it was necessary to conduct studies on a model of diet-induced obesity. Lantz et al. administered trodusquemine to mice with alimentary obesity and demonstrated suppressed appetite, reduced body weight in a fat-specific manner, and decreased plasma levels of insulin and leptin [181]. Subsequent enzymatic screening by the authors confirmed that trodusquemine selectively inhibits PTP1B. At the same time, insulin-stimulated phosphorylation of the insulin receptor and STAT3, direct targets of PTP1B, in HepG2 cells in vitro and in hypothalamic tissue in vivo was significantly increased. Thus, for the first time, it was shown that trodusquemine is an effective central and peripheral inhibitor of PTP1B.

This discovery was confirmed by studies of the role of the LMO4 protein, an endogenous inhibitor of PTP1B, in the hypothalamic nuclei [182]. It was found that the introduction of trodusquemine into the hypothalamus of LMO4-deficient mice restores central insulin signaling and improves the response of peripheral tissues to insulin [120]. Determination of the molecular mechanism of action of MSI-1436 prompted further research on its biological activity.

Despite the creation of effective, specific, and reversible low molecular weight inhibitors of PTP1B, the properties of the active site of the enzyme dictate that their molecules should be negatively charged (competitive inhibitors of PTP1B are phosphotyrosine mimetics [180]), which imposes restrictions on their bioavailability and limits their potential as drugs. Krishnan et al. revealed a new mechanism of allosteric inhibition of PTP1B, which is unique for trodusquemine [183]. The binding site located on the disordered C-terminal, the non-catalytic segment of PTP1B, as well as a second site close to the catalytic domain, were identified. The cooperative effect arising from the binding of the trodusquemine molecule to these centers blocks PTP1B in a catalytically inactive conformation [184].

#### 6.2.2. Anticancer Activity

Being an important regulator of cell signaling pathways, PTP1B also regulates the activity of kinase cascades associated with carcinogenesis, and, in particular, is a therapeutic target for HER2-positive cancers of the breast [177], lung [185], prostate [186], stomach [187], and colon [188]. PTP1B stimulates ErbB2-induced oncogenesis at the level of Ras/mitogen-activated protein kinase and PI3/protein kinase B signaling pathways. Additionally, its substrates are oncogenic proteins: receptor tyrosine kinases EGFR, insulin-like growth factor 1 receptor, platelet derived growth factor receptor, colony stimulating factor 1 receptor; protein tyrosine kinase c-Src, Jak2, Tyk2, FAK; transcription factors STAT5a and STAT5b; and adapter proteins p130Cas, Crk, p62Dok, β-catenin [185,189,190,191].

Fan et al. used trodusquemine to elucidate the important role of PTP1B as a negative regulator of BRK and IGF-1Rβ signaling in ovarian cancer cells [192]. In the already-mentioned study [183], trodusquemine showed the ability to suppress the HER2 signaling pathway by inhibiting tumor formation in xenografts and metastasis in the mouse model of NDL2 breast cancer. Thus, not only the effectiveness of PTP1B inhibition as a therapeutic strategy in breast cancer was confirmed, but also the potential of disordered protein segments as specific binding sites for therapeutic small molecules was shown.

#### 6.2.3. Antiatherogenic Properties

Cardiovascular disease is the most common cause of death in patients with type 1 or types 2 diabetes due to the development of endothelial dysfunction, accelerated atherosclerosis, and macrovascular complications [193,194,195]. Recent evidence suggests a strong relationship between atherosclerosis and insulin resistance due to impaired signaling through the insulin receptor [196,197,198]. In a mouse model of *LDLR*^−/−^ atherosclerosis, single and chronic administration of trodusquemine not only reduced body weight and obesity and improved glucose homeostasis but also attenuated the formation of atherosclerotic plaques [199]. This was accompanied by both a decrease in the level of total circulating cholesterol and triglycerides, as well as a decrease in the level of expression of the macrophage-1 chemoattractant protein and hyperphosphorylation of Akt/protein kinase B and AMPKα in the aorta. Thus, the possibility of using PTP1B inhibitors for the prevention and reversal of the development of atherosclerosis and the reduction of the risk of cardiovascular diseases was demonstrated for the first time.

#### 6.2.4. Regenerative Properties

The search for low molecular weight compounds with regenerative activity is a new and highly promising area of research [200]. Trodusquemine is the first-in-class regenerative drug prototype. Intraperitoneal administration of trodusquemine to adult zebrafish increased the rate of regeneration of the amputated caudal fin, which consists of bone, connective, cutaneous, vascular, and nervous tissue, and also increased the rate of myocardial regeneration. Intraperitoneal administration of trodusquemine to adult mice within 4 weeks after induction of myocardial infarction increased survival, improved heart function, decreased infarction size, decreased ventricular wall thickening, and increased cardiomyocyte proliferation. Doses effective in stimulating regeneration are 5–50 times lower than the maximum dose tolerated by humans. The shown safety and well-established pharmacological properties of trodusquemine underline the potential of this compound as a new treatment for myocardial infarction and other degenerative diseases [201,202].

#### 6.2.5. Anxiolytic Properties

Chronic stress can lead to the development of anxiety and affective disorders. The prevalence of these disorders and the lack of effectiveness of existing drugs necessitate the search for new methods of treatment [203]. Recently, the pathogenetic role of PTP1B in the development of anxiety disorders has been identified [204]. This opens up exciting opportunities for the use of PTP1B inhibitors as anxiolytics [205].

Stress disrupts LMO4-dependent inhibition of PTP1B, which in turn inhibits mGluR5, disrupting its mediated endocannabinoid production. Qin et al. used trodusquemine to confirm the central role of PTP1B in the development of chronic stress-induced anxiety [204]. They showed that treatment of F11 neuroblastoma cells with trodusquemine leads to increased tyrosine phosphorylation of mGluR5. Moreover, administration of the inhibitor to the amygdala, as well as systemic administration by intraperitoneal injection, attenuated the phenotypic manifestations of anxiety and schizophrenia-like behaviors in *LMO4* knockout mice [206]. Similar results were obtained after the introduction of lentiviral vectors expressing specific shRNA against PTP1B. In addition, they demonstrated that trodusquemine treatment inhibits the reduction of endogenous cannabinoid levels in the amygdala of stressed mice and reduces stress-induced anxiety.

### 6.3. Clinical Data

Phase 1 clinical trials have shown that squalamine is well tolerated in patients with advanced solid tumors [207,208]. When administered intravenously, a dose of 192–384 mg/m^2^/day did not cause toxic effects. Dose-limiting toxicity was observed at doses above 500 mg/m^2^/day as transient liver dysfunction (increased activity of hepatic transaminases and hyperbilirubinemia). Phase 1/2a clinical trials investigated the antitumor activity of a combination of 100–400 mg/m^2^/day squalamine with carboplatin and mg/m^2^/day paclitaxel in patients with stage IIIB–IV non-small cell lung cancer [209]. Thus, squalamine could be a valuable adjunct to the treatment of refractory cancers.

Squalamine lactate in the form of continuous intravenous infusion and eye drops has been clinically tested as a treatment for senile macular degeneration (abnormal growth of blood vessels in the choroid) [210]. Despite encouraging results and a good safety profile, trials of both drugs were suspended in 2007 and 2018 due to the introduction of monoclonal antibodies to VEGF into clinical practice. Later, in phase 2 clinical study in patients with macular edema caused by retinal vein occlusion, topical application of 0.2% squalamine in combination with intraocular administration of 0.5 mg ranibizumab (fragment of monoclonal antibodies to VEGF-A) restored vision more effective than ranibizumab monotherapy. The combination therapy was safe and well-tolerated [211]. A significant advantage of squalamine over anti-VEGF antibodies is the possibility of atraumatic topical application instead of intravitreal injections [212]. Despite these promising results, the phase 3 trial failed, presumably due to poor study design based on retrospective subgroup analysis [213,214].

Squalamine phosphate was orally administered in a pilot clinical study to patients with Parkinson’s disease (40 enrolled, 29 completed the dosing). The authors reported improved colon motility and significant amelioration of constipation along with some neurological and cognitive improvement. The effective dose ranged from 75 mg to 250 mg and was well tolerated, presumably due to low systemic bioavailability [22].

Currently, trodusquemine is the most-studied small molecule PTP1B inhibitor. The drug was originally developed by the compound’s discoverers, Magainin Pharmaceuticals, later renamed Genaera. It has successfully completed phase 1 clinical trials as a treatment for type 2 diabetes mellitus, showing good tolerability and pharmacokinetic profile in healthy individuals (NCT00509132, 2008), as well as in obese and type 2 diabetes patients (NCT00606112 and NCT00806338, 2009), and was planned to move to phase 2 trials. The financial difficulties of the developer prevented the implementation of these plans.

Trodusquemine is currently licensed to Depymed, which has launched phase 1 clinical trials for the treatment of HER-2 positive metastatic breast cancer (NCT02524951, 2017). The study was terminated in 2018 due to a lack of interest by the sponsor (Northwell Health, USA). Finally, the study of obstructive sleep apnea was announced by Angers University Hospital to evaluate the contribution of atherosclerosis and inflammation that can be ameliorated with trodusquemine (NCT04235023, 2020).

### 6.4. Synthetic Analogs of Squalamine and Trodusquemine and Structure-Activity Studies

The wide spectrum of pharmacological activity manifested by squalamine and trodusquemine prompted researchers to direct their efforts to structural analogs that are more accessible and can be scaled to industrial production.

In a study by Shu et al., a series of squalamine analogs were synthesized based on stigmasterol [34]. The 7α-hydroxyl substituent was either absent or replaced by 7β-hydroxyl. Analogs with 24-sulfate, 24-amino, and 24-hydroxy substituents were also synthesized to assess the importance of a functional design of the side chains for the manifestation of antimicrobial activity. All the derivatives obtained have significant antimicrobial activity, which indicates that the substitution of C7 and C24 for aminosterols does not play a decisive role in antibiotic properties. The most active compound, 210b, demonstrated MICs 1–2, 8, 8, 2 µg/mL against *S. aureus*, *E. coli*, *P. aeruginosa*, and *C. albicans*, respectively.

Similar patterns were revealed for a series of 3-amino- and polyaminosterol synthetic analogs of squalamine and trodusquemine lacking a sulfate group. The activity was shown to be highly dependent on the structure of the substituents at position C3, and one of the most active compounds comprised 3-(4-aminobutylamine)-moiety 117. For it, the minimum inhibitory concentrations against *S. aureus*, *E. faecalis*, *E. hirae* and *E. coli* were 6.25–25 μM [76].

Given the low availability of squalamine, numerous synthetically available analogs have been synthesized that exhibit broad-spectrum antibacterial activity with minimal inhibitory concentrations in the range of 2.5–40 μg/mL [33,34,76,81,110,215]. It was noted that analogs with a *tetra* ammonium polyamine fragment are more active than analogs with a shorter *tris* ammonium; while analogs with an axial (α)-hydroxyl substituent at C7 are more active than analogs with a corresponding equatorial (β)-hydroxyl group [70,216]. Derivatives with high activity against intracellular parasite *T. brucei*, the causative agent of African trypanosomiasis, and *L. donovani*, the causative agent of visceral leishmaniasis, have been described [85].

Recently, a series of cholestane squalamine analogs was described by Brunel et al. They lack sulfate moiety in the steroid side chain and, nevertheless, demonstrate similar squalamine activity against most common pathogens 124 [69]. Interestingly, an antibacterial and fungicidal activity comparable to squalamine was also observed for C7-spermidine analogs 134a and 134b [37], and 131 [78], suggesting that antibiotic properties of aminosterols depend on their amphiphilic nature and are not receptor-mediated.

Hydroxyl at C7 also seems to be dispensable for activity with compound **120** being more active than parent squalamine against *S. cerevisiae*, *C. albicans* and *E. feacalis* (MIC 6.25–12.5 µg/mL, but less active against *S. aureus* and *E. coli*) [74]. There is also a series of active derivatives against multi-resistant cocci, especially methicillin-resistant *S. aureus* at average concentrations of 2.5–5.0 μg/mL [75].

Another study focused on stereochemistry showed that 3β,5β-isomers have improved activity over α-counterparts with β-sperminyl-23,24-bisnor-5β-cholane 112 *S. aureus* having MIC value as low as 1 μg/mL against *S. aureus* ATCC6538P [73].

**Figure 9:**
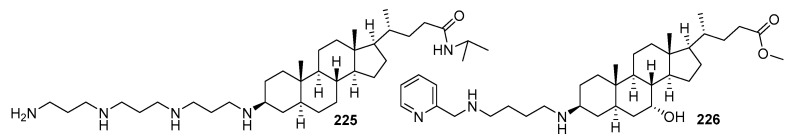
The structures of compounds **225** and **226**.

A series of compounds reported by Brunel et al. illustrated that the steroid backbone can tolerate amide functionality without compromising antibiotic activity [217]. Compounds exemplified by 225 (Figure 9) show high antibacterial and antifungal activty (MICs 2–8 μg/mL against and *P. aeruginosa* strains, MIC_50_ 0.5 μg/mL against *C. albicans*) and ability to potentiate activity of other antibiotics along with low cytotoxicity against CHO cells (CC_50_ 42 μg/mL). Conversion of squalamine zwitterion to amide-functionalized cations creates a valuable opportunity to overcome low intestinal absorption and inactivation by calcium ions.

Polyamine conjugates of stigmasterol showed diminished antibacterial activity (compound **170**, MIC 50 µg/mL against *S. aureus*) [88].

Bile acid-polyamine conjugates as synthetic ionophores and squalamine mimics demonstrated potent synergism with rifampin against many Gram-negative bacteria with spermine conjugate **195** being the most active (MIC 0.78–6.25 µg/mL against *E. coli*, *P. aeruginosa*, and *S. aureus*, although with a relatively low MHC of 12.5 µg/mL) [215]. A structurally relevant series of 3α-hydroxy-23,24-bisnorcholane spermidine and spermine carbamates was also reported [92]. In this case, steroidal backbone was replaced with carbamate bioisostere. Authors concluded that A,B-*cis* is superior to A,B-*trans* configuration. Carbamate 185 was the most potent against *S. aureus* and *P. aeruginosa* (MIC 0.78 µg/mL and 3.13 µg/mL, respectively; MHC 25 µg/mL). Conjugates of glycocholic acid with polyamine linker were also developed as modifiers of cholic acids intestinal and hepatic uptake to potentially mitigate first pass effect and improve the safety of hepatotoxic drugs [107].

Conjugate **84** as a synthetic ionophore showed comparable antibacterial activity to gentamicin against *S. aureus* with IC_50_ values of 4.12 µg/mL and was less effective against fungi with *Trichophyton mentagrophytes* being the most susceptible [69].

A polyaminosterol derivative of claramine [218], containing a spermine residue in the C6 position, similarly to trodusquemine, retains the property of selectively inhibiting PTP1B, practically without affecting the activity of the closest homolog, TC-PTP phosphatase. In neuronal cell culture, F11 both claramine and trodusquemine activated the insulin signaling pathway, increasing the phosphorylation of insulin receptor-β (IRβ), Akt, and glycogen synthase kinase 3 beta (GSK3β). Intraperitoneal administration of claramine or trodusquemine effectively restored glycemic control in diabetic mice in glucose and insulin tolerance tests. A single intraperitoneal injection of claramine or an equivalent dose of trodusquemine reduced food intake and led to weight loss in animals without increasing energy expenditure. Moreover, claramine proved to have pronounced antitumor activity in animal models of IL13Rα2 overexpressing cancers, including glioblastoma and colorectal carcinoma [219]. Claramine A1 also exhibits bactericidal activity against a wide range of Gram-positive and Gram-negative bacteria, including multiply resistant bacteria, and, as an adjuvant, restores the antibacterial activity of doxycycline against *P. aeruginosa* PAO1 and *E. aerogenes* EA28 [121].

The compound **226** (Figure 9), an orally bioavailable effective inhibitor of PTP1B (IC_50_ 100 nM versus 600 nM for MSI-1436) has also been described [220]. The MSI-1436-resistant mutant PTP1B L192A/S372P is inhibited by 226 with an IC_50_ of 1 μM. In addition, through several convincing experiments, the authors showed that 226 chelates the copper cation, which enhances its PTP1B-inhibitory activity. Compound 226 antidiabetic properties confirmed in an animal model of nutritional obesity [220].

Lou et al. has found that PDMS surfaces based on claramine derivative can be potentially useful for the elaboration of biomaterials preventing biofilm formation and addressing the issue of antibacterial resistance [221].

### 6.5. Ceragenins as Antibiotics

The discovery of ceragenins can be considered as a development of studies on the antibacterial activity of squalamine. As it was mentioned above (Section 3.11), ceragenins are positively charged polyamine derivatives of cholic acids that electrostatically interact with negatively charged phospholipids of bacteria, viruses, fungi, and protozoa and lead to an increase in fluidity, depolarization, and permeabilization of their membranes, which inhibits infectivity or results in bacteria death. Multi-resistant strains of bacteria that are susceptible to ceragenins include *S. aureus*, *S. pneumoniae*, *S. pyogenes*, *H. influenza*, *P. aeruginosa*, *N. meningitides*, *L. pneumophila* etc., *Candida*, *C. neoformans*, and *A. fumigatus* fungi, trypanosomes, as well as the vaccinia virus in 5 µM concentration [222,223,224].

These compounds exhibited antibacterial activity comparable or superior to polymyxin B against Gram-negative bacteria, and some also effectively permeabilize the outer membranes of Gram-negative bacteria [12,225,226].

**Figure 10:**
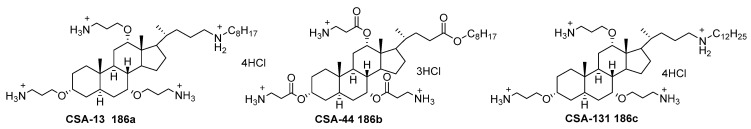
The structures of ceragenins **186a**–**c**.

The selectivity of the antibiotic action of the new compounds was assessed similarly to squalamine for its ability to induce lysis of eukaryotic cells. For example, 186a has a MIC for *P. aeruginosa* of 2 μg/mL and a minimum hemolytic concentration (MHC) of 29 μg/mL. In the presence of pluronic F-127, antibacterial activity of ceragenin 186a was only slightly decreased, but hemolytic activity was significantly inhibited. Ceragenin 186a exhibits bacterial killing activity against clinical isolates of *S. aureus*, including methicillin-resistant strains, *P. aeruginosa* present in cystic fibrosis sputa, and biofilms formed by different gram-positive and gram-negative bacteria [99,227]. These properties render ceragenins particularly useful in orthopedic medicine as antibiotics and implant coatings [228,229]. Silicone coating incorporating 186a developed by Williams et al. [230] proved to be biocompatible, safe, and effective against MRSA biofilms in vivo [231,232]. Another example is contact lenses made with covalently bound 186b or 186c releasing polymers that resist bacterial colonization with *S. aureus* or *P. aeruginosa* for 15–30 days (Figure 10) [233].

The selectivity of action is explained by the ability to bind to lipid A, specific for prokaryotic membranes, which was confirmed using amphiphilic steroids labeled with a fluorophore [105]. Ceragenin 186a builds into a phospholipid bilayer which results in increased fluidity and destabilization [234]. Similar to other aminosterols, ceragenins elicit bacterial membrane permeabilization and act synergetically with other antibiotics and antimicrobials, which was confirmed for colistin, tobramycin, ciprofloxacin, LL-37, lysozyme, and lactoferrin [99,235]. Importantly, ceragenins elicit different gene responses in *E. coli* as compared to cationic antimicrobial peptides, which is associated with a lower level of resistance [236]. Another important benefit of ceragenins is their improved activity in cystic fibrosis sputum. It was shown that ceragenin 186a is significantly less sensitive to extracellular polyanions that compromise the antibacterial activity of cationic antibacterial peptides [97]. CSA-13 186a has potent antibacterial and antibiofilm activity against *Achromobacter spp*. [237] and *P. aeruginosa* strains isolated from cystic fibrosis patients (MIC_90_ 2 µg/mL) and acts synergetically with colistin, a polymyxin antibiotic [235]. Moreover, colistin-resistant and chlorhexidine-resistant Gram-negative bacteria strains remain susceptible to ceragenins 186b and 186c (Figure 10) [238,239]. On top of this, ceragenin 186c exceeds the activity of antimicrobial peptides against preformed bacterial and fungal-bacterial biofilms [240,241], and 186a also stimulates cell migration which facilitates wound healing [242]. These results were confirmed in the porcine model of burn wounds with ceragenin 186b [243]. Another hard-to-fight pathogen, *B. subtilis* spores, is also sensitive to CSA-13 186a treatment [244,245].

Moreover, CSA-17 effectively eradicates drug-resistant clinical isolates of *H. pylori* (MBC 0.275–8.9 μg/mL) and even retains activity in simulated gastric juice containing pepsin and mucins that inactivates peptide-based antibiotics [144]. Recently it was shown that ceragenins may be used to overcome bacterial resistance to carbapenems. NDM-1 carbapenemase-producing strains of *E. coli*, *E. cloacae*, and *K. pneumoniae* are susceptible to 186a–186c with MICs as low as 1–2 µg/mL [246]. Ceragenins 186a and 186c are also promising drugs against carbapenem-resistant *A. baumannii* strains [247], improving antibacterial activity of LL-37 peptide against drug-resistant *E. coli* strains isolated from patients with urinary tract infections [248], they retain activity against multi-drug resistant strains of *K. pneumoniae* [249].

Ceragenins possess high fungicidal activity against a broad spectrum of pathogenic fungi, e.g., 186a showed MIC in 0.5–4 µg/mL against *C. albicans* and *Candida spp.* [250,251], including fluconazole-resistant strains [252]. The mechanism of action of ceragenins against fungi is not precisely defined but as derivatives of cholic acid that mimic the morphology of natural antimicrobial peptides, they are expected to act similarly, causing damage and dysfunction of the plasma membrane. Ceragenins 186a and 186c have stronger candidacidal activity than natural peptide and omiganan against all tested fluconazole-resistant yeast cells as well as against young and mature biofilms [250].

In an attempt to further improve bactericidal activity and biocompatibility of 186a several nanoparticle formulations were developed. The MNP-CSA-13 nanoparticles demonstrate dual benefits: a decrease of ceragenin hemolytic activity and an increase of antimicrobial properties in body fluids [101]. MNP approach improved the antibacterial effect of CSA-13 against methicillin-resistant *S. aureus* and *P. aeruginosa* [253]. Ceragenins 186 and 186c attached to MNPs exceed the antibacterial activity of LL-37 or metronidazole against *B. fragilis*, *P. acnes*, and *C. difficile* and prevented formation of their biofilms [254]. Furthermore, MNP-CSA-13 prevents *Candida spp*. Biofilm formation which is relevant for the treatment of fungal infections in immunocompromised patients [255]. Silver nanoparticles conjugated with ceragenin, or cationic antimicrobials (CSA-SNPs) hold potential as Gram-positive selective antimicrobial [99]. Formulation of 186c in poloxamer micelles prevents damage to ciliated tissues while retaining bactericidal activity against established biofilms [256].

Later several studies revealed that the membrane-permeabilizing capability of 186a is linked not only to antimicrobial, but also anticancer properties [257]. Ceragenin 186a induces cell cycle arrest and apoptosis in wild-type and p53 null mutant HCT116 colon cancer cells at 5 µg/mL concentration [258]. MCF-7 and MDA-MB-231 breast cancer cells are also susceptible both to free CSA-17 and CSA-17-loaded magnetic nanoparticles unlikely to antimicrobial LL-37 peptide, which is protumorigenic [259]. Similar to antibacterial studies, ceragenins immobilized on metal nanoparticles demonstrate synergistically improved therapeutic potential against cancer cells. MNP-186a nanoparticles are also active against DLD-1 colon cancer cells [260]. In these cases, 186a was a carrier to internalize MNP and induce intracellular oxidative stress followed by apoptosis. Anticancer activity of MNP-186c at a 10 µg/mL dose was further confirmed against several colon and lung cancer cell lines [261].

To sum up the data of this section, we should conclude that aminosterols have several principal benefits over commonly used antibacterial drugs. Ceragenins hold promise to fight important human pathogens that are either multi-drug resistant or might become resistant to currently used antibiotics in a near future. The development of second-generation ceragenins and their nanoformulation in liposomes or nanoparticles demonstrated that a combination of efficacy against various bacteria and fungi, even in established biofilms, with biocompatibility and stability in vivo, is indeed possible. The mechanism of antibiotic action relies on physicochemical interaction with phospholipids of cellular membranes. Accordingly, there is no evidence that resistance to aminosterols may emerge since they do not engage protein targets and are not subjected to enzymatic inactivation or active efflux. In comparison with antimicrobial peptides, which share a similar mechanism of action, aminosterols are less expensive to produce, resistant to proteolytic degradation, which permits oral administration and retain their activity in biological fluids. Unlike peptides, they are thermally stable enough to permit autoclave sterilization and use in implant coatings or contact lenses, and even facilitate wound healing and reparation of bone fractures.

The second promising application is linked to antiangiogenic activity for the treatment of neoplastic diseases. Widely used cytostatic agents have intrinsic toxicity towards normal cells, which results in immunodepression, gastrointestinal disorders, and alopecia. Furthermore, high proliferation rates permit the expansion of mutant cancer cells that evade cytostatic therapy. Targeting vascularization of tumors instead is prone to the development of resistance and proved to be tolerable in clinical trials. Choroidal neovascularization is also effectively managed by topical squalamine administration that provides an alternative to traumatic injections of anti-VEGF antibodies in age-related macular edema and similar conditions.

Last but not least, trodusquemine and its synthetic analogs represent unique orally available and brain penetrant allosteric inhibitors of PTP1B. There are no clinically approved drugs targeting this important enzyme, thus aminosterols hold promise to be first-in-class drugs that may relieve the burden of such important diseases such as type 2 diabetes mellitus, obesity, cancer, neurodegenerative, cardiovascular, and psychiatric disorders, including depression and schizophrenia. Further clinical trials are warranted to confirm their safety and efficacy and to ultimately provide benefit to wide cohorts of patients (Table 1).

## 7. Terpene- and Triterpene-Based Polyamine Derivatives

**Scheme 35:**
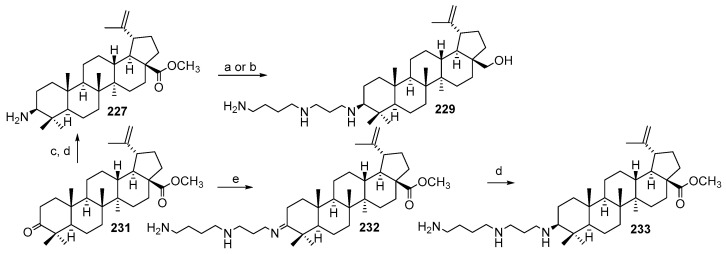
Reagents and conditions: (**a**) NC(CH_2_)_3_N(Boc)(CH_2_)_3_I 228, 95% HCOOH, LiAlH_4_, THF, 66 °C; (**b**) H_2_N(CH_2_)_4_N(Boc)(CH_2_)_2_CHO 230, benzene, Ti(O*i*Pr)_4_, 80 °C; (**c**) NH_2_OH HCl, Py, 114 °C; (**d**) NaBH_3_CN, NH_4_OH, 15% TiCl_3_, MeOH, 25 °C; (e) H_2_N(CH_2_)_3_NH(CH_2_)_4_NH_2_, Ti(O*i*Pr)_4_, 80 °C, benzene.

As was mentioned above, the chemistry and biological activity of steroid polyamines had a strong impact on the synthesis of terpenoid-based polyamine derivatives that started in the first decade of the 21st century. Literature analysis shows that the main group of terpenoid polyamines is obtained on the basis of triterpenic acids. For example, two syntheses of C3 conjugates with spermidine have been realized. The interaction of methyl 3β-amino-3-deoxybetulinoate 227 with *tert*-butyl-*N*-(4-cyanobutyl)-*N*-3-(iodopropyl)-carbonate 228 led to betulin conjugate 229 in a 24% yield. In a yield of 21%, compound **229** was synthesized by reductive amination of derivative 227 with *tert*-butyl-*N*-(4-aminobutyl)-*N*-3-(oxopropyl) carbonate 230 in the presence of NaBH_3_CN. Conjugate 233 was obtained in a total yield of 34% by the reaction of methyl betulonoate 231 with *N*-(3-aminopropyl)-1,4-diaminobutane in the presence of Ti(O*i*Pr)_4_ to form imine 232 and its subsequent reduction with NaBH_3_CN (Scheme 35) [262].

**Scheme 36:**
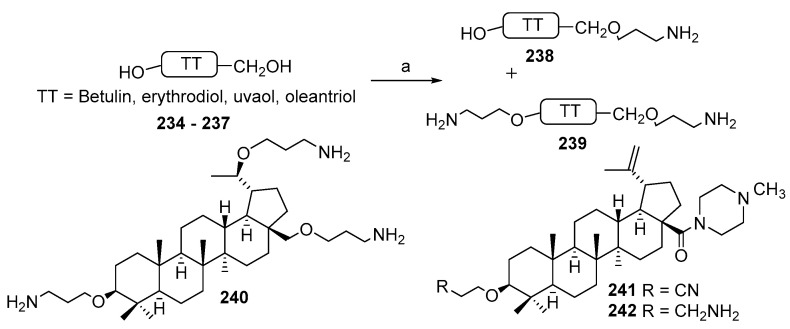
Reagents and conditions: (a) 1. CH_2_=CHCN, TEBAC, dioxane, 40% KOH, 25 °C, 26–36 h; 2. H_2_, Raney-Ni, MeOH, 100 °C, 100 atm, 19 h.

Another approach included the reaction of cyanoethylation of triterpene alcohols. Interaction of diols 234–237 with acrylonitrile in dioxane resulted in mixtures of 28-mono- and 3,28-biscyanoethyl ethers with the prevalence of the latter (Scheme 36). As a result of catalytic hydrogenolysis of cyano-derivatives, aminopropoxy-modificants of betulin, erythrodiol, uvol, and oleantriol of type 238 and 239 have been synthesized [263]. Aminopropoxytriterpenoids have proven to be highly active anticancer agents, inhibiting the growth of colon cancer, leukemia, breast cancer, and melanoma. 3,28-*bis*-Aminopropoxy-erythrodiol showed high antitumor activity against five transplanted mouse tumors [263,264]. 3,28-*bis*-Aminopropoxy-betulin was found to be a potent micromolar inhibitor of yeast α-glucosidase and simultaneously inhibit endosomal reticulum α-glucosidase, rendering it potentially capable to suppress tumor invasiveness and neovascularization in addition to the direct cytotoxicity [265]. Using the described approach, 3β,20*R*,28-*tri*-(3-aminopropoxy)-betulin 240 [266,267] and 2-cyanoethoxy- 241 and 3-aminopropoxy-betulinic *N*-methylpiperazinylamide 242 were synthesized and showed highly cytotoxic activities towards non-small cell lung, colon, breast, ovarian, leukemia, renal, melanoma, prostate and CNS cancer cells [264,268].

**Scheme 37:**
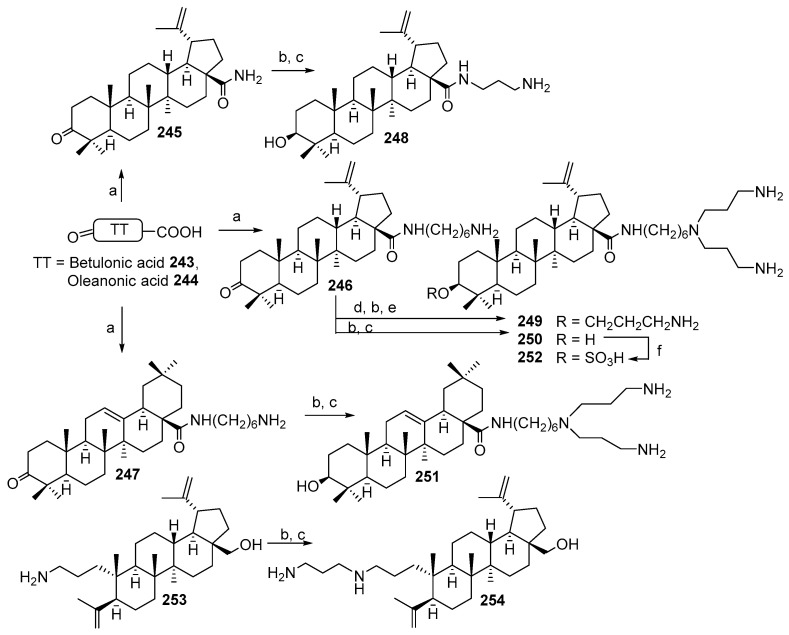
Reagents and conditions: (**a**) 1. (COCl)_2_, CHCl_3_, 2 h, 25 °C; 2. NH_3_ or NH_2_(CH_2_)_6_NH_2_, Et_3_N, CHCl_3_, 60 °C, 3 h; (**b**) CH_2_=CHCN, 40% KOH, dioxane, TEBAC, 14 h; (**c**) LiAlH_4_, THF; (**d**) NaBH_4_, *i-*PrOH, 0 °C, 2 h; (**e**) H_2_, Raney-Ni, MeOH, 100 °C, 100 atm, 8 h; (**f**) H_2_SO_4_, Ac_2_O, Py, 55 °C, 1 h, then 0 °C, 15 min.

Triterpenoids with alkane polyamine fragments in the C28 side chain were synthesized on the basis of betulonic and oleanonic acids 243, 244. The reaction of cyanoethylation of the terminal amino group of triterpene carboxamides 245–247 led to the formation of mono- or *bis*-*N*-propionitriles, the reduction of which afforded 3-aminopropylamine derivatives 250 and 251 (Scheme 37). Compound 250 was converted to partially soluble sulfate 252 [266,269]. In a similar route, aminopropyl group was introduced into the structure of A-*seco*-3-aminobetulin 253 with the formation of 3-aminopropylamino-derivative 254 [270].

**Scheme 38:**
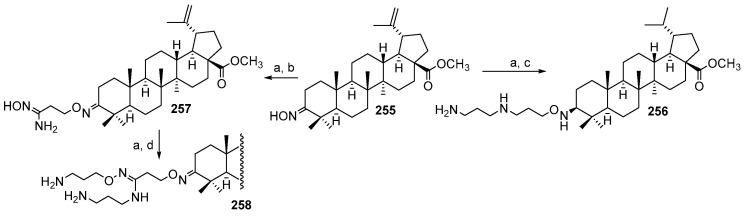
Reagents and conditions: (**a**) CH_2_=CHCN, dioxane, 40% KOH, 25 °C, 2 h; (**b**) NH_2_OH HCl, NaHCO_3_, *i*-PrOH, 77 °C, 8 h; (**c**) NaBH_4_, BF_3_ Et_2_O, THF, 65 °C, 6 h; (**d**) H_2_, Raney-Ni, MeOH, 100 °C, 100 atm, 9 h.

Cyanoethylation of methyl betulonoate oxime 255 led to 3-cyanopropoxy-amino derivative, the following reduction with diborane afforded methyl 3-deoxy-3β-(3-aminopropoxyamino)-20(29)dihydrobetulinoate, the terminal NH_2_-group of this compound was cyanoethylated again and reduced to form a polyamine 256. Stepwise interaction of 255 with acrylonitrile and hydroxylamine led to compound **257**, and repeated cyanoethylation, and catalytic hydrogenolysis afforded derivative 258 (Scheme 38) [270].

**Scheme 39:**
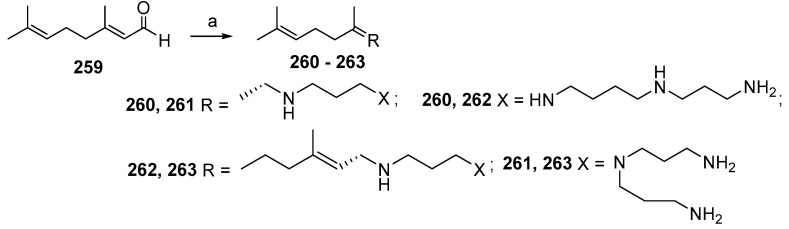
Reagents and conditions: (**a**) R-NH_2_, Ti(O*i*Pr)_4_ (1 eq.), MeOH, 20 °C, 12 h; NaBH_4_ (2 eq.), H_2_O, 0 °C, 2 h.

A next series of derivatives was synthesized on the basis of monoterpenoids. Thus, the synthesis of isoprene polyamines 260–263 was reported by the interaction of citral 259 with polyamines (Scheme 39) [271,272]. The study of the activity of derivatives 260–263 together with the antibiotic doxycycline against the resistant strain of *P. aeruginosa* showed that compound **262** destabilizes the outer membrane and inhibits the outgoing cell pumps, which facilitates easy penetration of the antibiotic into the bacterium. Thus, they created an opportunity for the rejuvenation of forgotten antibiotic molecules with the help of “escort molecules” to improve their action [271]. They were assayed against clinical isolates and multi-drug-resistant strains. One of these compounds was able to decrease the MIC of doxycycline on the reference strain, efflux pump overproducers, and clinical isolates of *P. aeruginosa*, to the susceptibility level. Similar results were obtained using chloramphenicol as the antibiotic. Membrane permeation assays and real-time efflux experiments were used to characterize the mechanism of doxycycline potentiation. The results showed that the selected compound strongly decreases the efficiency of glucose-triggered efflux associated with a slight destabilization of the outer membrane. According to these data, targeting natural resistance may become an interesting way to combat MDR pathogens and could represent an alternative to already devised strategies.

**Scheme 40:**
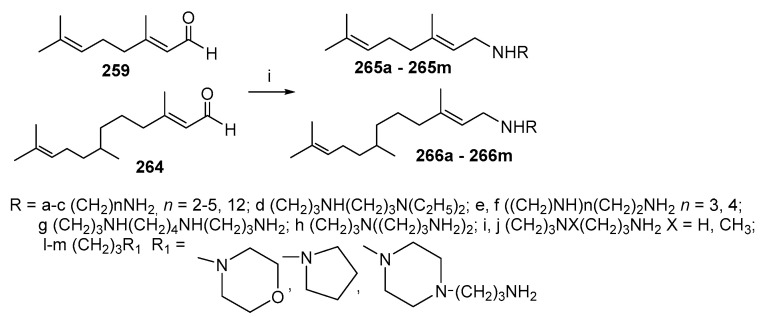
Reagents and conditions: (**i**) R-NH_2_, Ti(O*i*Pr)_4_ (1 eq.), MeOH, 20 °C, 12 h; NaBH_4_ (2 eq.), −78 °C, 2 h.

Monoterpene derivatives 265 and 266 (Scheme 40), were successfully evaluated for their in vitro antibiotic enhancer properties against resistant Gram-negative bacteria of four antibiotics belonging to four different families. The mechanism of action against *E. aerogenes* of one of the most efficient of these chemosensitizing agents was precisely evaluated by using fluorescent dyes to measure outer-membrane permeability and to determine membrane depolarization. The weak cytotoxicity encountered led to performing an in vivo experiment dealing with the treatment of mice infected with *S. typhimurium* and affording preliminary promising results in terms of tolerance and efficiency of the polyaminoisoprenyl derivative 266h-doxycycline combination [273].

**Figure 11:**
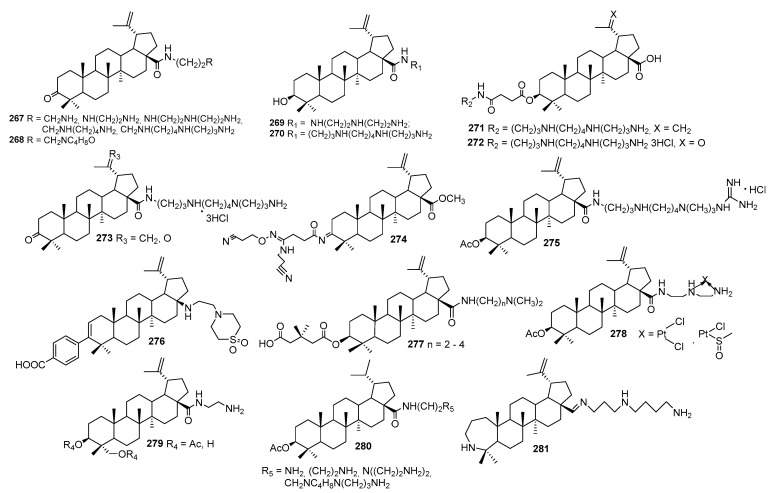

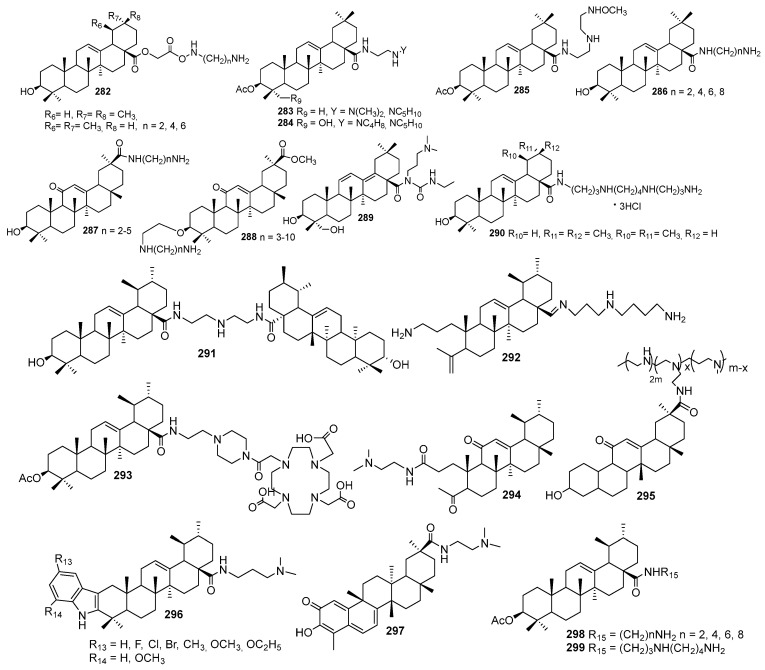
The structures of triterpene conjugates with polyamines **267** [265,275], **268** [281], **269** [265], **270**, **271** [300], **272**, **273**, **290** [298], **275**, **299** [299], **276** [294,303], **277** [258], **278** [259], **279** [260], **280** [295,299], **281**, **292** [302], **282** [289], **283** [274], **284** [304], **285** [287], **286** [291], **287** [284], **288** [277], **289** [285], **291** [283], **293** [296], **294** [280], **295** [297], **296** [279], **297** [274].

Among triterpene-based polyamines, the most representative group is presented by triterpenic conjugates with linear and cyclic diamines 267–299 (Figure 11) [263,264,265,267,274,275,276,277,278,279,280,281,282,283,284,285,286,287,288,289,290,291,292,293,294,295,296,297,298,299]. The reactions involved both native acids and their semi-synthetic derivatives. For almost all compounds, data on biological properties were obtained, mainly on cytotoxicity against cancer cells, antiviral, antibacterial, antidiabetic, and antifungal activity. It is interesting to note that conjugates of betulinic, oleanolic, ursolic, and platanic acids with spermine at the C28 or C3 positions through a succinate spacer exhibited not only antimicrobial and antitumor activity [298,300], but also self-assembled into J-type fibrous systems in aqueous media, and also form supramolecular networks, which opens up many possibilities for the use of such structures for drug delivery systems in serum or other body fluids [298,301]. Triterpene aldimines with spermidine were found to be promising antibacterial agents against both Gram-positive and Gram-negative bacteria [302]. Amide BMS-955176, derived from betulin in seven steps, is an effective antiretroviral drug in phase 2b clinical trials [294,303]. Lupane carboxamides, conjugates with diaminopropane, triethylenetetramine and branched methyl 3-cyanoethylated polyamine betulonoate showed high cytotoxic activity against most of the tested cancer cell lines with the lowest GI_50_ 1.09 µM. Betulonic acid diethylentriamine conjugate showed partial activity against methicillin-resistant *S. aureus* and the fungi *C. neoformans* [265].

**Figure 12:**
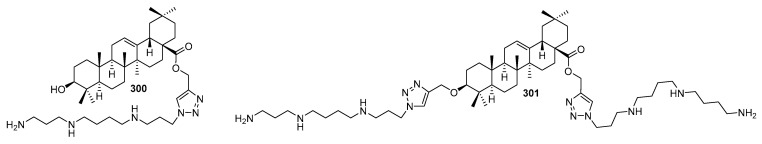
Conjugates of oleanolic acid with spermine **300** and **301**.

Conjugates of oleanolic acid with spermine **300** and **301** were studied for the characteristics of their oleanolic acid backbone that is a conformationally rigid and convenient chiral building block for preparing functional soft materials. However, besides their supramolecular characteristics, conjugates displayed high cytotoxicity with a range IC_50_ 0.8–3.7 µM (Figure 12) [305].

**Figure 13:**
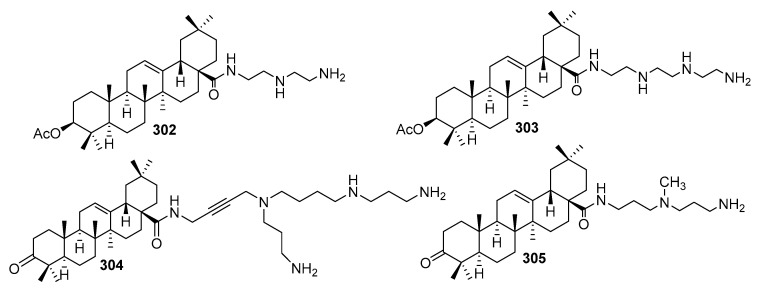
Conjugates of oleanolic acid with diethylenetriamine **302**, triethylenetriamine **303**, oleanonic acid conjugate with spermine spacered through propargylamide **304**, and *N*-methyl-norspermidine **305**.

Oleanolic acid conjugate with diethylenetriamine 302 (Figure 13) demonstrated high inhibitory activity against *C. trachomatis* with chemotherapeutic index 8 and >8. Compounds 302 and 303 exhibited remarkable activities against the NCI-60 subpanel (GI_50_ ranges from 0.18 to 2.21 μM) exceeding the activity of sorafenib with compound **302** as a lead (GI_50_ 0.17 μM for melanoma LOX IMVI) [306]. A series of oleanolic acid derivatives holding oxo- or 3-*N*-polyamino-3-deoxy-substituents at C3 as well as carboxamide function at C28 with different long chain polyamines have been synthesized and showed good antimicrobial activities against Gram-positive *S. aureus*, *S. faecalis* and *B. cereus* (MIC values from 3.125 to 200 µg/mL) and Gram-negative *E. coli*, *P. aeruginosa*, and *S. enterica* (MIC ranging from 6.25 to 200 µg/mL) [307]. The testing of ability to restore antibiotic activity of doxycycline and erythromycin at a 2 µg/mL concentration in a synergistic assay showed that oleanonic acid conjugate with spermine spacered through propargylamide 304 led to a moderate improvement in terms of antimicrobial activities of the different selected combinations against both *P. aeruginosa* and *E. coli*. The study of mechanism of action of the lead conjugate 305 presenting a *N*-methyl norspermidine moiety showed the effect of disruption of the outer bacterial membrane of *P. aeruginosa* PA01 cells.

## 8. Conclusions

**Figure 14:**
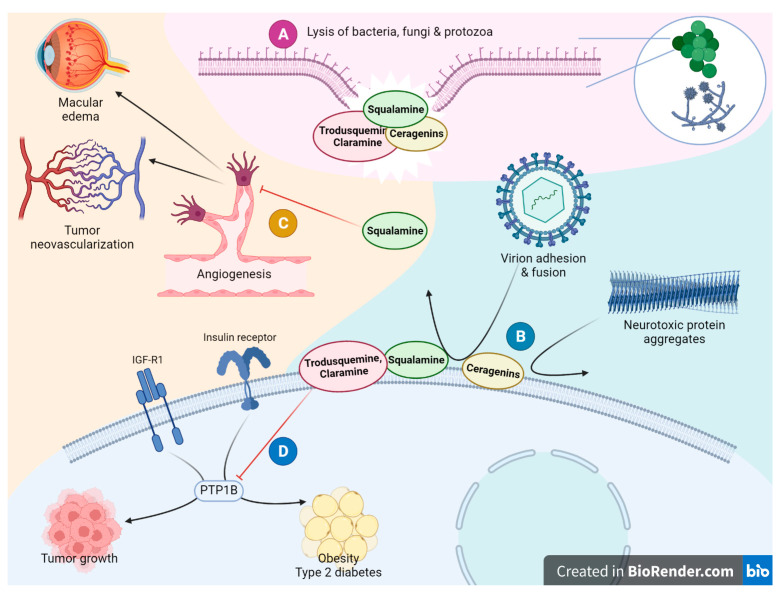
Polyamine steroids exhibit diverse biological activity via several distinct mechanisms: permeabilization of bacterial and fungal membranes kills pathogens (**A**); modification of eukaryotic cell membranes renders them resistant to virions and misfolded proteins (**B**); inhibition of pathological angiogenesis hampers development of macular edema and tumors (**C**); PTP1B inhibition improves insulin sensitivity, decreases food intake and directly suppresses cancer cells proliferation (**D**).

Squalamine and trodusquemine isolated from the dogfish shark *Squalus acanthias* at the turn of 1990–2000 were involved in the systematic chemical and clinical investigations. Due to the need for significant amounts for biological tests, different synthetic approaches were suggested for their preparation from available steroids and cholic acids. In vitro and in vivo studies of squalamine showed a broad-spectrum bactericidal antibiotic activity against both Gram-positive and Gram-negative bacteria, and antiviral activity against RNA- and DNA viruses, and an inhibition of pathological angiogenesis associated with cancer and retinopathy. Trodusquemine and its synthetic analogs represent unique orally available and brain penetrant allosteric inhibitors of PTP1B, and these aminosterols hold promise to be first-in-class drugs that may relieve the burden of such important diseases including type 2 diabetes mellitus, obesity, cancer, neurodegenerative, cardiovascular, and psychiatric disorders (Figure 14).

From the beginning of the first decade of the 21st century, the chemistry and biological activity of steroid polyamines had a strong impact on the synthesis of terpenoid-based polyamine derivatives. The study of isoprene polyamines together with the doxycycline against the resistant strain of *P. aeruginosa* revealed the derivative that destabilizes the outer membrane and inhibits the outgoing cell pumps, which facilitates easy penetration of the antibiotic into the bacterium, thus creating an opportunity for the rejuvenation of forgotten antibiotic molecules with the help of “escort molecules” to improve their action. Triterpenic conjugates with spermine, spermidine, triethylenetetramine, other linear and cyclic di- and polyamines as well as branched aminopropoxy-derivatives have been synthesized. For almost all compounds, data on cytotoxicity against cancer cells, antiviral, antibacterial, antidiabetic, and antifungal activities were obtained. Among them, conjugates of several triterpenic acids with spermine exhibited not only antimicrobial and antitumor activity, but also formed self-assembled systems and supramolecular networks in aqueous media, which opens up many possibilities for the use of such structures for drug delivery systems in serum or other body fluids.

## Figures and Tables

**Table 1:** Therapeutic profiles of squalamine, trodusquemine, claramine, and ceragenins.

Primary Target	Pharmacological Action	Evidence Level			
		Squalamine	Trodusquemine	Claramine	Ceragenins
**Phospholipid membranes**	Antibacterial	In vitro MIC 1–8 μg/mL: *E. coli*, *P. aeruginosa*, *S. aureus*, *S. faecalis*, *P. vulgaris*, *K. pneumoniae* [38], *A. baumannii* [132]; MIC 0.5–1 μg/mL *Methanobrevibacter* spp. [130,131]; in animal studies as monotherapy or as sensitizer for conventional antibiotics against resistant strains of *S. aureus*, *E. coli*, *P. aeruginosa*, *E. aerogenes*, *K. pneumoniae* [103,128,142]	In vitro MIC 1–4 μg/mL: *S. aureus*, *P. aeruginosa*, *C. albicans* [7]	In vitro MIC 2–16 μg/mL: *E. coli*, *E. aerogenes*, *E. cloacae*, *P. aeruginosa*, *K. pneumoniae*, *A. baumannii* [120], and as an adjuvant for doxycycline against *P. aeruginosa* PAO1 and *E. aerogenes* EA28 [121]	In vitro MIC 2–4 μg/mL: *E. coli*, *S. aureus*, *S. pneumoniae*, *S. pyogenes*, *H. influenza*, *P. aeruginosa*, *N. meningitides*, *K. pneumoniae*, *L. pneumophila*, *B. subtilis* [226,227,229,230,231,239,242,248,249,250,251,252,253]; in animal studies as sensitizer for conventional antibiotics [99,228,229,235,243]
	Antifungal	In vitro MIC 12.6–25 μM: *C. albicans*, *C. krusei*, *C. glabrata*, *A. fumigatus*, *A. niger*, *Fusarium* spp.) and protozoa (*P. caudatum*) [127]			In vitro MIC in 0.5–4 µg/mL: *Candida* spp., *C. neoformans*, *A. fumigatus* [245,254,255,256]
	Antiviral	In vitro: Dengue virus, hepatitis B, yellow fever, herpesviruses [146,147]; in animal models: yellow fever, eastern equine encephalitis, cytomegalovirus [30]	In vitro: HIV [167]		In vitro: vaccinia virus [224]
Misfolded proteins	Neuroprotective	In vitro: alpha-synuclein, amyloid-beta [148,149]; in animal models of Parkinson’s disease [15,153]; phase 2 clinical trilas of squalamine phosphate for Parkinson’s disease [22,158]	In vitro: alpha-synuclein, amyloid-beta, HypF-N [119,152,153,154,155,157] In animal models of Parkinson’s and Alzheimer’s diseases (presumably PTP1B is also involved) [157,159,160,161]		
NHE3 and mitogen signalling	Antiangiogenic	As an adjuvant to cytostatic drugs in animal tumor models [14,152,164,165,166]; as a monotherapy in animal retinopathy models [167,168,169]; squalamine lactate as monotherapy or in combination with mAb to VEGF in phase 2 clinical trilas for age-related macular edema [210,211,212,213,214]; combinatorial therapy in phase 2 clinical trilas for non-small cell lung cancer [210,211,212]			
PTP1B	Antidiabetic via insulin and leptin receptors signalling		As monotherapy in diabetic ob/ob and db/db mice [173,174,175] and DIO mice [181,220]; as monotherapy in phase 1 clinical study for type 2 diabetes mellitus (NCT00606112, NCT00806338)	As monotherapy in diabetic CaMK2aCre/LMO4flox mice [218]	
	Anticancer via growth factor receptors signalling		As monotherapy in animal models of solid tumors [180,186,188,189,190,191,192,193,194,195]; as monotherapy in phase 1 clinical study for HER-2 positive metastatic breast cancer (NCT02524951)	As monotherapy in animal models of glioblastoma and colorectal carcinoma [219]	
	Atherosclerosis		In animal models of *LDLR*^−/−^ atherosclerosis [199]; phase 1 study has been announced		
	Anxiolytic		In animal models of anxiety and schizophrenia [205,206]		
	Regenerative		In animal models of trauma and myocardial infarction [201,202]		

## Data Availability

No new data were created or analyzed in this study.

## Abbreviations

Akt	RAC-alpha serine/threonine-protein kinase
AMPK5′	adenosine monophosphate-activated protein kinase
BOP	*N*-(benzenesulfonyl)-*L*-prolyl-*L*-*O*-(1-pyrrolidinylcarbonyl)-tyrosine sodium salt
BRK	BRICK1 subunit of SCAR/WAVE actin nucleating complex
DAT	dopamine active transporter
DAST	(diethylamino)sulfur trifluoride
(DHQD)_2_PHAL	hydroquinidine 1,4-phthalazinediyl diether
EEDQ	2-ethoxy-1-ethoxycarbonyl-1,2-dihydroquinoline
FAK	focal adhesion kinase
FRET	förster or fluorescence resonance energy transfer
GTPase	guanosine triphosphate hydrolase
HOBt	1-hydroxybenzotriazole
HypF-N	*N*-terminal domain of the *E. coli* HypF carbamoyltransferase
IL13Rα2	interleukin-13 receptor α2
Jak2	Janus kinase 2
LDLR	low density lipoprotein receptor
LMO4	LIM domain only 4
MBC	minimum bactericidal concentration
MCP-1	macrophage-1 chemoattractant protein
MDR	multidrug-resistant
mGluR5	metabotropic glutamate receptor 5
MHC	minimum hemolytic concentration
MNP	magnetic nanoparticles
mRNA	messenger RNA
MRSA	methicillin-resistant *S. aureus*
MECBS	2-methyl-CBS-oxazaborolidine
NDM-1	new Delhi metallo-beta-lactamase
NHE	sodium-hydrogen exchanger
PADS	petromyzonamine disulfate
PDMS	Polydimethylsiloxane
PI3K	phosphoinositide 3-kinase
PEI	Polyethyleneimines
PTP1B	protein tyrosine phosphatase 1B
PTSA	4-toluenesulfonamide
shRNA	small hairpin RNA
SNP	silver nanoparticle
STAT	signal transducer and activator of transcription
TBHP	*tert*-butyl hydroperoxide solution
TEMPO	2,2,6,6-tetramethylpiperidine 1-oxyl
Tyk2	non-receptor tyrosine-protein kinase 2
TMCS	trimethylsilyl chloride
VEGF	vascular endothelial growth factor
